# Formin Is Associated with Left-Right Asymmetry in the Pond Snail and the Frog

**DOI:** 10.1016/j.cub.2015.12.071

**Published:** 2016-03-07

**Authors:** Angus Davison, Gary S. McDowell, Jennifer M. Holden, Harriet F. Johnson, Georgios D. Koutsovoulos, M. Maureen Liu, Paco Hulpiau, Frans Van Roy, Christopher M. Wade, Ruby Banerjee, Fengtang Yang, Satoshi Chiba, John W. Davey, Daniel J. Jackson, Michael Levin, Mark L. Blaxter

**Affiliations:** 1 School of Life Sciences, University of Nottingham, Nottingham NG7 2RD, UK; 2 Center for Regenerative and Developmental Biology, and Department of Biology, Tufts University, Medford, MA 02155, USA; 3 Institute of Evolutionary Biology, University of Edinburgh, Edinburgh EH9 3JT, UK; 4 Department for Biomedical Molecular Biology, Ghent University, and Inflammation Research Center (IRC), VIB, 9052 Ghent, Belgium; 5 Wellcome Trust Sanger Institute, Wellcome Trust Genome Campus, Hinxton, Cambridge CB10 1SA, UK; 6 Community and Ecosystem Ecology, Division of Ecology and Evolutionary Biology, Graduate School of Life Sciences, Tohoku University, Aobayama, Sendai 980-8578, Japan; 7 Department of Geobiology, University of Göttingen, Göttingen 37077, Germany; 8 Edinburgh Genomics, School of Biological Sciences, University of Edinburgh, Edinburgh EH9 3JT, UK

## Abstract

While components of the pathway that establishes left-right asymmetry have been identified in diverse animals, from vertebrates to flies, it is striking that the genes involved in the first symmetry-breaking step remain wholly unknown in the most obviously chiral animals, the gastropod snails. Previously, research on snails was used to show that left-right signaling of Nodal, downstream of symmetry breaking, may be an ancestral feature of the Bilateria [1, 2]. Here, we report that a disabling mutation in one copy of a tandemly duplicated, diaphanous-related formin is perfectly associated with symmetry breaking in the pond snail. This is supported by the observation that an anti-formin drug treatment converts dextral snail embryos to a sinistral phenocopy, and in frogs, drug inhibition or overexpression by microinjection of formin has a chirality-randomizing effect in early (pre-cilia) embryos. Contrary to expectations based on existing models [3, 4, 5], we discovered asymmetric gene expression in 2- and 4-cell snail embryos, preceding morphological asymmetry. As the formin-actin filament has been shown to be part of an asymmetry-breaking switch in vitro [6, 7], together these results are consistent with the view that animals with diverse body plans may derive their asymmetries from the same intracellular chiral elements [8].

## Highlights

• Animals tend to be outwardly symmetric but internally are asymmetric
• Unlike other animals, snails show inherited variation in asymmetry
• We found that both snails and frogs use a common gene to define left and right
• Asymmetry is probably an ancient and conserved property of cells and animals

## Teaser

Davison et al. have discovered a cell scaffolding protein in snails and frogs that controls body asymmetry, either the direction a snail shell coils, or whether a frog heart is placed to the left or right. Body asymmetry in animals, including humans, likely arises from a highly conserved, intrinsic asymmetry of the cell.

## Results and Discussion

Bilaterian animals are more or less symmetrical about the midline that divides left and right, but internally most organs are asymmetric in location or shape. How is symmetry broken during development if the “right” and “left” sides are essentially arbitrary? A longstanding model posits that a chiral “F molecule” is orientated relative to the anteroposterior and dorsoventral axes [9]. This asymmetric molecular reference then determines left-right (LR) differentiation at the cellular and organismal level.

Given the importance of chiral patterning in the three bilaterian superphyla, Deuterostomia, Ecdysozoa, and Lophotrochozoa, a continuing problem is a lack of knowledge of the first symmetry-breaking steps in the Lophotrochozoa, even though the first described locus that reverses the whole body structure of an animal was from the pond snail *Lymnaea* [10, 11]. Recently, commonalities between different species have been discovered [12, 13, 14, 15]. For example, in both vertebrates (Deuterostomia) and snails (Lophotrochozoa), *nodal* and *pitx* encode key signaling molecules required for the establishment of LR asymmetry, suggesting that these genes may have been used in the last common ancestor of Bilateria, but lost in Ecdysozoa [1, 2, 16]. However, neither *nodal* nor *pitx* is the earliest symmetry-breaking determinant in snails, ultimately limiting a knowledge of whether this represents deep conservation or convergent use of the same genes.

Within Lophotrochozoa, snails are unique in that they exhibit genetically tractable, natural variation in chirality [17] and so may aid in understanding of the establishment and conservation of LR asymmetry. Here, we use genetics, genomics, and pharmacological inhibition to show that the *Lymnaea stagnalis* chirality gene is a scaffolding component of the cytoskeleton. We present strong evidence that this same molecule is one component of an early chiral cytoskeletal structure that is involved in the earliest symmetry-breaking steps across the Bilateria.

### A Gene that Is Associated with Chirality in Pond Snails

**Figure 1:**
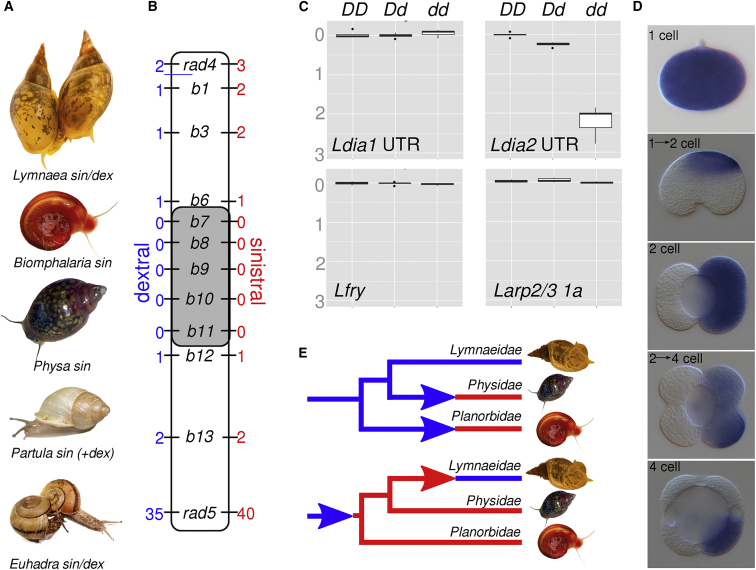
Mapping the Formin Gene, Maternal Expression, and Evolution of Chirality (A) The snail genera used in this study (image credits: *Lymnaea* [E. de Roij], *Biomphalaria* and *Physa* [creative commons], *Partula* and *Euhadra* [A.D.]). (B) 3,403 offspring were used to infer the recombination breakpoints that bound the *D* locus. Numbers of mapped recombinants for 1,507 sinistral (*dd*) snails are shown on the right and for 1,896 dextral (*DD* or *Dd*) on the left. The sinistral mutation must be between loci b6 and b12 (shaded), a region that spans 267 kb (not to scale). (C) Boxplots show normalized relative quantities (NRQs), on log scale, of quantitative real-time PCR assays of transcripts of three candidate genes and one control (*Larp2/3 1a*) in single-cell egg samples from dextral homozygote (*DD*), dextral heterozygote (*Dd*), and sinistral recessive homozygote (*dd*) individuals. Significant differences in expression were detected for *Ldia2* only (*DD*:*dd*, p = 0.002; *DD*:*Dd* and *Dd*:*dd*, p = 0.004). (D) WMISH of maternal *Ldia* transcripts in early, dextral *L. stagnalis* embryos. (E) Schematic showing two hypotheses for the evolution of chirality in three snail families (dextral = blue; sinistral = red). Either sinistrality evolved once from a dextral ancestor, with the ancestral Lymnaeid reverting to dextral (bottom), or sinistrality evolved twice (top). See also Table S1 for the mapping data; Figure S3 for further WMISH and quantitative real-time PCR data; and Figure S2C for the full snail phylogeny.

The gastropod mollusc *L. stagnalis* is naturally variable in left-right asymmetry, outwardly visible in the chirality of the spiral shell, and under the control of a single maternally expressed locus. In *L. stagnalis* (Figure 1A), maternal *D* alleles dominantly determine a clockwise (“dextral”) twist in offspring [18, 19]. Specifically, during the third cleavage in dextral embryos, four micromeres simultaneously emerge from four macromeres and twist clockwise (“spiral deformation”; [19]). In homozygous recessive (*dd*) sinistral embryos the four micromeres initially emerge neutrally, without rotation, with a later counterclockwise twist taking place during furrow ingression [19]. These differences are presaged by the orientation of metaphase-anaphase spindles. In dextral embryos, micromere spindle orientation is chiral (“spindle inclination”; [19]), while in sinistral embryos, the spindles are positioned radially, such that they do not exhibit chirality.

Previously we defined a ∼0.4 Mb region of the 1 Gb *L. stagnalis* genome that must contain the chirality locus [18]. To identify the chirality-determining gene, we generated two new resources: a sequenced bacterial artificial chromosome (BAC) clone walk across the chirality locus interval and draft genome sequences of *DD* homozygote and *Dd* heterozygote individuals. The BAC clone walk was oriented using three-color fiber fluorescent in situ hybridization (FISH) (Figure S1). We used the offspring of a large cross to recombination breakpoint map the position, orientation, and haplotype origin (*D* or *d*) of each BAC clone relative to the original restriction associated DNA sequencing (RAD-seq) markers (rad4 and rad5) and the chirality locus *D* (Table S1). The chirality locus was located between markers b6 and b12, a region of 267 kb (Figure 1B). We predicted genes across the scaffolded BAC walk assembly and mapped *DD* and *Dd* whole-genome sequencing data to the assembly to identify haplotype-specific variation.

Only six genes were in perfect linkage with *D* in our mapping cross, all contained on a single BAC scaffold: lysosomal pro-x carboxypeptidase (*Lprcp*), furry (*Lfry*), a tudor domain-containing protein (*Ltud*), a major facilitator superfamily domain-containing protein (*Lmfsd*), and a pair of tandemly duplicated diaphanous-related formin genes (*Ldia1* and *Ldia2*; Figure S2A). In sinistral (*dd*) snails, *Ldia2* was found to contain a homozygous, single base deletion in the 5′ of the coding region that causes a frameshift. The mutation was confirmed by cloning and sequencing *Ldia* transcripts from both sinistral and dextral snails (KU341302–KU341305). No disabling mutations were discovered in the coding sequences of the other genes, including the adjacent paralog *Ldia1*.

Maternal expression of genes was assessed by quantitative real-time PCR in single-cell embryos from *DD*, *Dd*, and *dd* mothers. Of the six candidate genes, only *Ldia2* showed significant differences in expression associated with genotype (Figures 1C and S3A). *Ldia2* transcripts were readily detected in dextral embryos from *DD* mothers. However, in sinistral embryos from *dd* mothers, *Ldia2* transcript levels were ∼0.6% that of *DD* embryos, whereas in embryos from heterozygous *Dd* mothers, they were ∼50%. These differences in expression are consistent with frameshifted *Ldia2*^sin^ transcripts being degraded by nonsense mediated decay.

The tandem duplication perhaps explains why a *Ldia2*^sin^ mutation is not simply lethal [20, 21, 22], in that *Ldia1* and *Ldia2* may have overlapping roles in embryonic development, albeit with some specialized function: while we found that mRNAs for both *Ldia1* and *Ldia2* were present in equal quantities in the dextral 1-cell embryo, *Ldia2* was ∼3-fold enriched in 1-cell dextral embryos relative to somatic tissues, whereas *Ldia1* was ∼10-fold depleted (∼30-fold difference overall).

### Asymmetric Expression of Maternal Genes Precedes Asymmetric Morphology

Expression of candidate loci was examined in early *L. stagnalis* embryos using whole-mount in situ hybridization (WMISH; [23]). The first two embryonic cleavages in *L. stagnalis* are equal, so that the four macromeres that are formed from the first two cleavages are indistinguishable [3, 4, 5], until contact between the third quartet of micromeres induces one of the macromeres to become the future D blastomere (but see 24]). We expected that mRNA transcripts would initially be equally distributed between the four macromeres. Surprisingly, however, we found that *Ldia* mRNA was asymmetric at the 2-cell stage and largely confined to one macromere by the 4-cell stage (Figure 1D), with *Lfry* sometimes showing an asymmetric pattern, albeit less striking, and with more variation between individuals (Figure S3B).

While we do not know whether the *Ldia2*-positive macromere is the D blastomere, individual macromeres must have an identity as early as the two-cell stage, contrary to the expectation based on accepted models [3, 4, 5] of development in equal cleaving snails. This also further emphasizes the general point that asymmetry is determined very early and intracellularly—asymmetry of molecules precedes visible morphological asymmetry, although not necessarily in a causative manner (see [4, 25] for comparison with unequal cleaving embryos). The results also suggest an explanation for the reduced viability of sinistral embryos [20, 21, 22]—as chiral rotation of micromeres in sinistrals is sometimes incomplete or error prone, inviability may be caused by a conflict in identity between individual cells. Further investigation is necessary, but the sinistral embryos seem to show more generalized, less obviously asymmetric staining, consistent with this explanation (perhaps because of reduced transport on actin microfilaments, [4]) (Figure S3B).

### Pharmacological Inhibition of Formin in Early Snail Embryos Mimics the Sinistral Phenotype

As transgenic approaches are not yet established in *L. stagnalis*, and microinjection is usually lethal [22], we tested formin involvement in chirality using SMIFH2, an FH2 domain inhibitor [26]. SMIFH2 prevents formin nucleation and processive elongation of filamentous actin by decreasing the affinity of formin for the barbed end. Micromolar concentrations of SMIFH2 disrupt the formation of formin-dependent, but not Arp2/3 complex-dependent, actin cytoskeletal structures [26].

**Figure 2:**
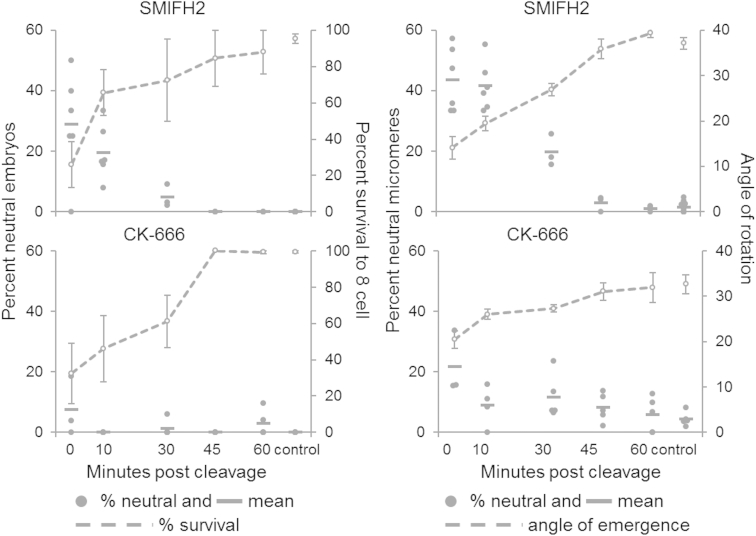
Impact of Drug Treatment upon 4-Cell Snail Embryos When applied shortly after the second cleavage had completed, both SMIFH2 and CK-666 reduced the proportion of embryos that survived to the 8-cell stage (left-hand graphs). Following SMIFH2 treatment (top left), a high proportion of the viable embryos emerged neutrally, without a chiral twist. In contrast, the proportion of neutral embryos following CK-666 treatment (bottom left) was low. Both drugs reduce the angle of rotation as the micromeres emerge (right-hand graphs). Mean values for each experiment and SE are shown. See also Table S2 and Movie S1.

**Figure 3:**
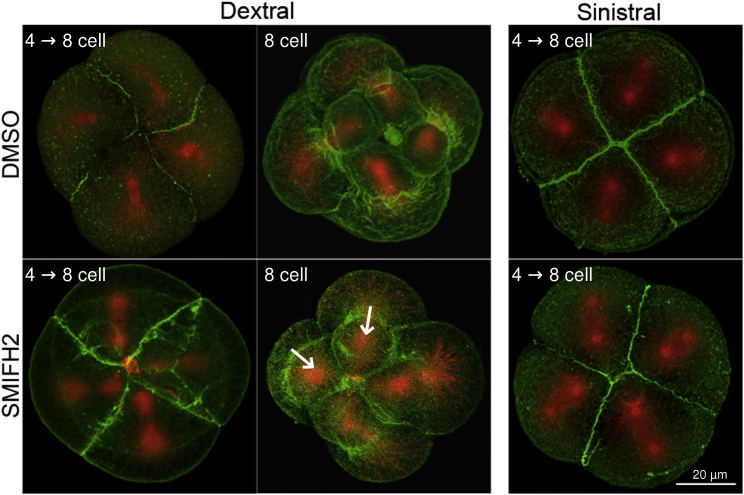
Tubulin and Actin Staining of Control and Drug-Treated Embryos Embryos were fixed and stained with Cy3-b-tubulin (red) and 488-phalloidin (green) to highlight the spindle microtubules and filamentous actin, respectively. DMSO-treated embryos predominantly showed spindle inclination (left image, 4-cell stage), with the micromeres usually emerging with a dextral twist (right image, 8-cell stage). A minority of SMIFH2 treated embryos had mitotic spindles that showed a radial orientation (left image), an arrangement that was not observed in DMSO control dextral embryos. In the SMIFH2 right-hand image (8-cell stage), the top middle and middle left micromeres are emerging neutrally (arrows), with the other two showing a partial rotation. Addition of SMIFH2 did not influence spindle orientation in 4-cell DMSO control or sinistral embryos, with spindles typically showing a radial orientation.

When 100 μM SMIFH2 was added to genetically dextral 4-cell embryos shortly after completion of the second cleavage, relatively few embryos (∼30%–60%) reached third cleavage at ∼80 min (Figure 2; Table S2). However, in ∼25%–35% of those that did, all four micromeres emerged neutrally, with no chiral twist (SMIFH2 treated at 0 min, n = 6 experiments, 274 embryos, compared to n = 11 control experiments, 219 embryos; p < 0.001, *U* = 5.5, Mann-Whitney U), with up to ∼45% of individual micromeres emerging neutrally (Figure 2). SMIFH2 treatment of dextral embryos thus phenocopies normal sinistral embryos. We also visualized spindles during the third cleavage of *L. stagnalis* by indirect immunofluorescence with anti-β-tubulin antibody. In line with previous findings [19], control dextral embryos showed the characteristic spindle inclination, especially in the latter stages of mitosis. In SMIFH2-treated dextral embryos, the spindles were more frequently radially symmetric, resembling untreated sinistral embryos (Figure 3).

To rule out a non-specific effect of SMIFH2, we compared the effect of another inhibitor of actin assembly, CK-666, which acts specifically on Arp2/3 complex-dependent actin patches [27] and not on formin-dependent actin cables. CK-666 also had a lethal effect when applied to genetically dextral early four-cell embryos and also tended to reduce the average angle of emergence of micromeres. However, an achiral phenotype was observed in rather few CK-666-treated embryos (Figure 2; CK-666 treated 0 min, n = 3 experiments, 196 embryos, compared to n = 5 control experiments, 190 embryos; p = 0.107, *U* = 2.5, Mann-Whitney U). Therefore, the differential effects of SMIFH2 and CK-666 suggest that formin-mediated actin assembly may be a critical factor in determining chiral cleavage orientation between the second and third cleavages.

In SMIFH2-treated, genetically dextral embryos that continued to develop following neutral emergence of micromeres (i.e., like sinistrals), the direction of the subsequent twist was dextral, rather than sinistral (6/6; Movie S1). The individual micromeres of genetic sinistrals also sometimes twisted dextrally (∼0 to 4% in our experiments; the fourth time-lapse in Movie S1 shows an embryo in which all four micromeres twist dextrally after neutral emergence; see also [22]). This later twist is also actin dependent [19], which suggests dextrality may be the default pathway, independent of FH2 domain function.

### A Sinistral Ancestral Pond Snail?

We used genomic and transcriptomic resources, and new sequences, to explore links between diaphanous formin and chiral evolution. First, we found that the chromosomal region that contains the *L. stagnalis* chirality locus is deeply conserved, exhibiting synteny of *dia*, *fry*, and *tud* between *L. stagnalis*, *Biomphalaria glabrata* (planorb snail, sinistral cleaving), and *Capitella telata* (polychaete annelid, dextral cleaving). Second, while the *dia* duplication is also evident in *Lymnaea trunculata*, and so must pre-date its divergence from *L. stagnalis*, single-copy *dia* genes in *B. glabrata* and *Physa acuta* (both sinistral snails: Figure 1A) are more similar to *Ldia1* than *Ldia2* (Figure S2B). The *B. glabrata dia gene* and *Ldia1* also share a non-repetitive UTR element, absent from *Ldia2*. Thus, *Ldia2* is likely the derived paralog, which may have evolved specific function in the embryo, and for which loss leads to a sinistral phenotype.

We mapped chirality onto a new phylogeny of the Hygrophila (Figure S2C). The predominantly dextral Lymnaeidae and the sinistral Physidae cluster together, so either sinistrality evolved once, with a sinistral ancestral Lymnaeid subsequently reverting to dextral, or else, sinistrality evolved on two separate occasions (Figure 1E). Both explanations are equally parsimonious. However, only the first is consistent if the duplication was involved in enabling dextrality in the Lymnaeidae.

As no natural variation in chirality has been described in *Biomphalaria* or *Physa*, it is difficult to further test the function of *dia* in relation to copy number. Instead, we sampled *dia* orthologs in two other chirally variable snail genera, *Euhadra*, a Japanese land snail, and *Partula*, an endangered species from Polynesia (Figure 1A; [28, 29]). In both of these genera, chirality was not associated with variation in a single-copy *dia* that was recovered (Figure S2). Molecular understanding of chiral variation in these species is therefore likely to reveal additional components of the LR asymmetry pathway, including variants in genes that enable chiral evolution without negative pleiotropic effects upon fitness.

### Formin Also Regulates LR Patterning in the Frog

A popular model of LR symmetry breaking in vertebrates relies on chiral flow of extracellular fluid during neurulation. However, this mechanism cannot be universal as many phyla lack the ciliated structures required or achieve correct LR patterning prior to the ciliated structure differentiation (reviewed in [8, 14, 15]). Inherent asymmetry in the cytoskeleton could provide an ancient, well-conserved mechanism used by vertebrate embryos at the earliest stages of development to initiate the LR pattern and instruct the entire body plan [8, 30, 31]. We investigated whether formin inhibition in early embryos, before the neurula ciliary flow, could affect chirality in the vertebrate model *Xenopus laevis*.

**Figure 4:**
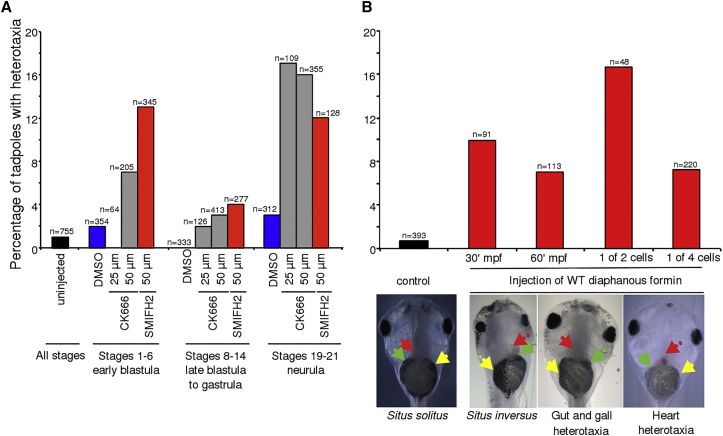
Effect of Drug Treatment and Microinjection of Overexpressed Formin on Chirality in the Frog (A) Embryos were treated with DMSO, CK-666, or SMIFH2 at the concentrations indicated, allowed to develop, and scored for visceral organ chirality at stage 45. (B) Embryos were injected into the animal pole with mRNA encoding mouse *dia1* formin and scored for visceral organ *situs* at stage 45. Images: Examples of organ *situs* for experimental microinjection with wild-type mouse *dia1* mRNA. The control shows a wild-type (*situs solitus*) tadpole, ventral view, demonstrating the normal arrangement of the stomach (yellow arrowhead), heart apex (red arrowhead), and gall bladder (green arrowhead). Heterotaxic tadpoles (ventral view) resulting from formin overexpression show reversal of all three organs, i.e., *situs inversus*; the gut position and looping and gall bladder; or the heart. See also Table S3 and Figure S4.

SMIFH2 and CK-666 drug treatments were carried out with *X. laevis* embryos at different stages of development and scored by measuring heterotaxia (independent organ LR inversion) in tadpoles (Figure 4; Table S3). In early embryos (stages 1–6), during which the cytoskeleton instructs LR patterning [30], treatment with 50 μM SMIFH2 had a strong effect on LR patterning (13% heterotaxia, X^2^, p < 0.001), with a smaller but significant effect with CK-666 (7% heterotaxia, X^2^, p < 0.001), whereas embryos treated at neurula stages (stages 19–21), when ciliary flow is present, showed a strong effect for both 50 μM SMIFH2 (12%, X^2^, p < 0.001) and 50 μM CK-666 (16%, X^2^, p < 0.001). Treatments spanning late blastula to gastrula (stages 8–14) had a reduced (3%–4%) but still significant effect (Table S3) on organ heterotaxia, showing that the efficacy of early treatment cannot be due to remnant drug persisting to cilia stages.

For a more specific gain-of-function test of formin function in *Xenopus*, mouse *dia1* mRNA was injected into the animal pole of frog embryos and organ *situs* assessed at stage 45. Similar to the in vitro finding that the spontaneous counterclockwise alignment of actin bundles can be reversed by overexpression of alpha-actinin [7], we found that overexpression of *dia1* resulted in a high and significant proportion of heterotaxia, whether injected 30 or 60 min post-fertilization, or in one of two or four cells (Figures 4 and S4). In addition, targeted injection into the dorsal left (DL) or ventral right (VR) blastomeres at the 4-cell stage showed that while significantly different from uninjected controls (X^2^, p < 0.001), there is no significant difference between DL and VR in the effect on heterotaxia (n = 85 DL, 5% heterotaxia; n = 135 VR, 9% heterotaxia; p = 0.75 Student’s t test), which would be expected if the effect were at the point of ciliary flow, as the left side of the embryo is required for ciliary flow affecting LR patterning [32].

### Formin, an F Molecule?

The implication of a key cytoskeletal protein in LR patterning of both molluscan and vertebrate embryos is consistent with a view of asymmetry as a highly conserved, ancient property in which diverse body plans leverage asymmetry from the same intracellular chiral elements. Bilaterian LR asymmetry may be dependent upon the physical orientation of the actin cytoskeleton, which, by exerting mechanical stresses on the cell, results in helical rotation [6, 7]. While multiple elements potentially contribute to the establishment of this asymmetry, formins may have pivotal roles in coordinating functions that depend upon both the actin and microtubule cytoskeleton [7, 30, 33, 34]. Pond snails are now an experimentally tractable, comparative model in which to integrate understanding of the action of downstream patterning genes, such as *nodal*, and the dynamics of cellular interaction and movement in the embryo to generate handedness.

## Author Contributions

Conceptualization, A.D., M.L., and M.L.B; Methodology and Investigation, A.D. and C.M.W. (phylogenetics); Methodology and Investigation, H.F.J., A.D. (quantitative real-time PCR), J.M.H., and A.D. (snail microscopy and pharmacology); Methodology and Investigation, D.J.J. (WMISH), R.B., and F.Y. (FISH, with help from A.D. and M.M.L.); Methodology and Investigation, A.D. and M.M.L. (BAC walk, with BioS&T); Methodology and Investigation, G.S.M. and M.L. (laboratory - frog); Methodology and Investigation, A.D. and S.C. (Japan fieldwork); Methodology and Investigation, A.D., C.M.W., D.J.J., G.D.K., J.W.D., M.L.B., P.H., and F.V.R. (bioinformatics); Writing – Original draft, A.D. and M.L.B.; Writing – Review & Editing, A.D., C.M.W., D.J.J., G.S.M., G.D.K., H.F.J., J.M.H., M.M.L., M.L., and M.L.B. Supervision and Data Analysis, A.D., M.L., and M.L.B; Discovered the mutation, A.D. The order in which G.S.M. and J.M.H. appear in the author list was decided by a “best of three” coin toss.

## Accession Numbers

The accession numbers for sequences in the INSDC are: PRJEB11470 (all libraries), ERS1022296 (genomic libraries), and ERS935814 (BAC libraries). GenBank accession numbers are KU341240–KU341321. Dryad accession is http://dx.doi.org/10.5061/dryad.r4342.

## Supplemental Information

Document S1. Supplemental Experimental Procedures, Figures S1–S4, and Tables S1–S3

Movie S1. Time-Lapse of Third Cleavage, Related to Figures 1 and 2 In embryos from genetically dextral mothers, the micromeres twist dextrally as they emerge. In embryos from genetically sinistral mothers, the micromeres emerge neutrally, then twist sinistrally after emergence. In SMIFH2-treated embryos from genetically dextral mothers, the micromeres frequently emerge neutrally, then twist dextrally after emergence. Occasionally, in embryos from genetically sinistral mothers, the micromeres emerge neutrally, as normal, but the later twist is dextral.

Document S2. Article plus Supplemental Information
