# Supplementary material for: Formin Is Associated with Left-Right Asymmetry in the Pond Snail and the Frog
Source: Curr Biol. 2016 Mar 7;26(5):654–60. doi: 10.1016/j.cub.2015.12.071 (PMC4791482; doi:10.1016/j.cub.2015.12.071)
Supplement: Document S1. Supplemental Experimental Procedures, Figures S1–S4, and Tables S1–S3 [file mmc1.pdf]

Current Biology, Volume *26*

Supplemental Information

Formin Is Associated with Left-Right Asymmetry in the Pond Snail and the Frog

Angus Davison, Gary S. McDowell, Jennifer M. Holden, Harriet F. Johnson, Georgios D. Koutsovoulos, M. Maureen Liu, Paco Hulpiau, Frans Van Roy, Christopher M. Wade, Ruby Banerjee, Fengtang Yang, Satoshi Chiba, John W. Davey, Daniel J. Jackson, Michael Levin, and Mark L. Blaxter

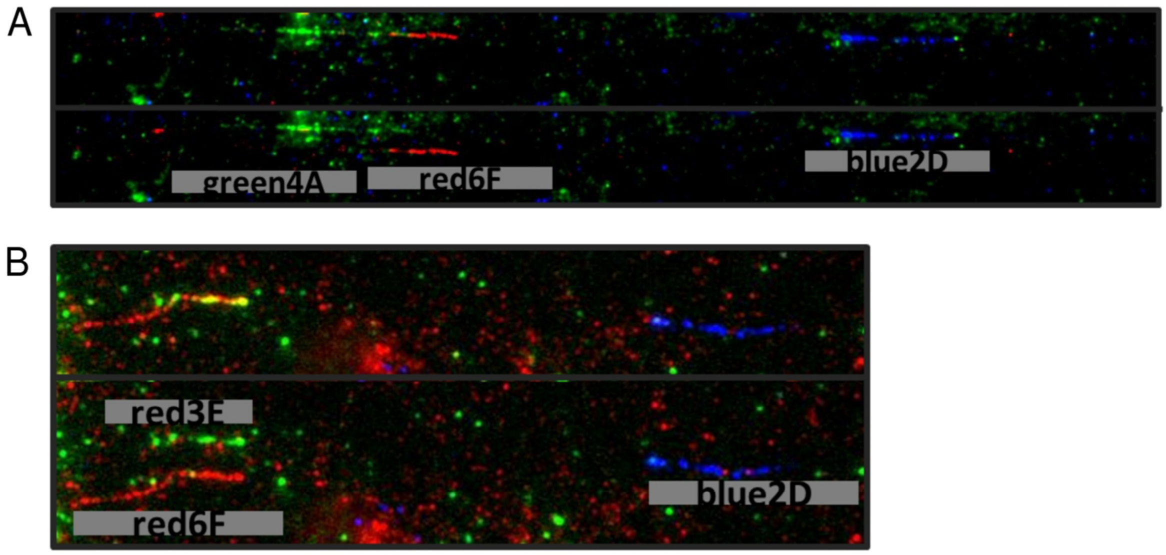

Figure S1. Related to Figure 1. Using fibre FISH on BACs that bound the chirality locus to infer orientation.

The gap between the hybridisation signal from BAC clones containing loci rad4 (BACs red6F, 129 kb; green4A, 110 kb; red3E (colour green, 112 kb) and rad5 (blue2D, 97 kb) is at least 4 to 5 times the length of a BAC clone, confirming that previously reported. In the lower parts of both (A) and (B)**,** the red and green BACs have been shifted for clarity.

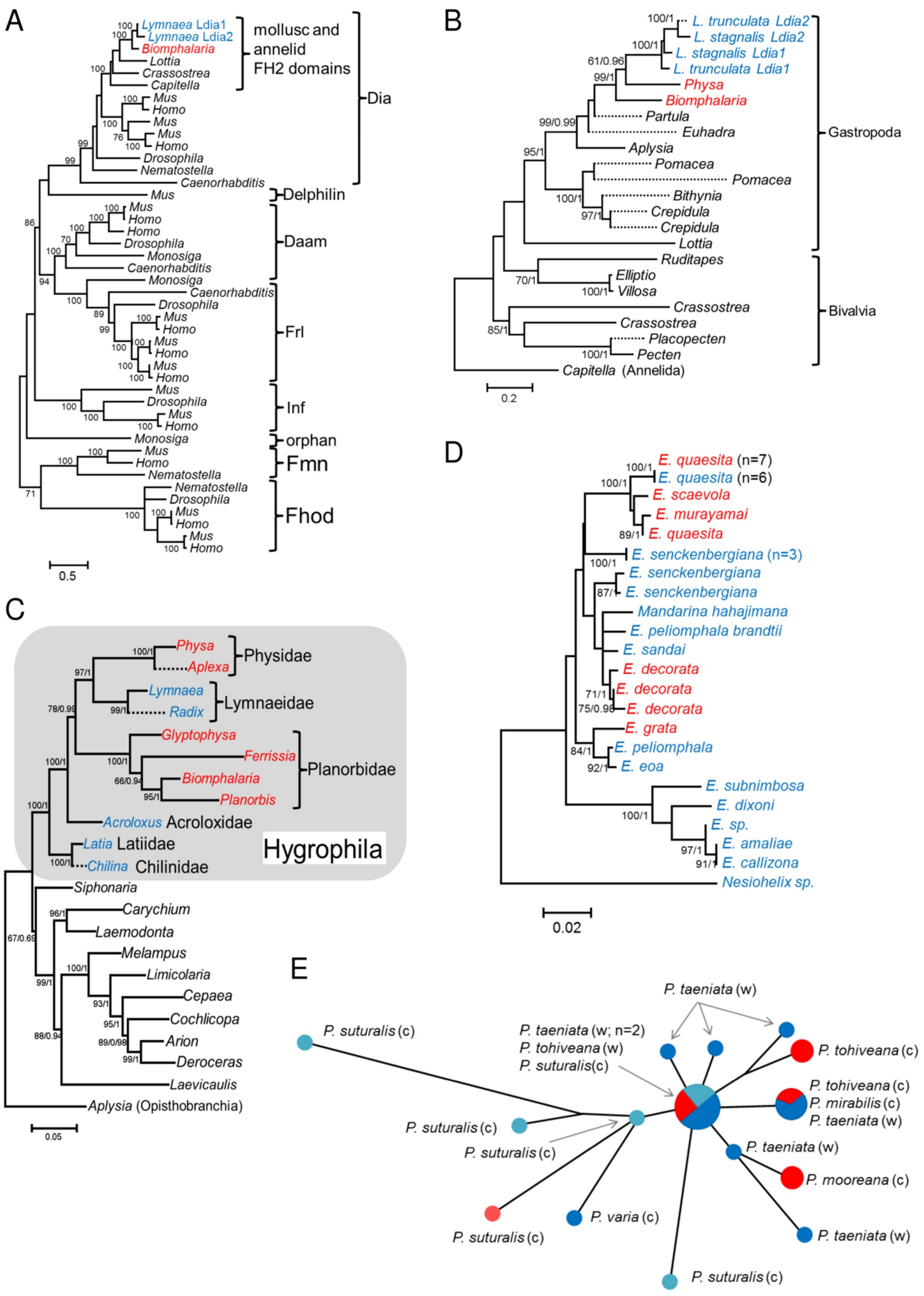

A
Lymnaea Ldia1
Lymnaea Ldia2
Biomphalaria
Lottia
Crassostrea
Capitella
mollusc and
annelid
FH2 domains
Dia
Mus
Homo
Drosophila
Nematostella
Caenorhabditis
Delphilin
Daam
Monosiga
Frl
Inf
orphan
Fmn
Fhod
0.5
B
L. trunculata Ldia2
L. stagnalis Ldia2
L. stagnalis Ldia1
L. trunculata Ldia1
Physa
Biomphalaria
Partula
Euhadra
Aplysia
Pomacea
Bithynia
Crepidula
Lottia
Gastropoda
Ruditapes
Elliptio
Villosa
Crassostrea
Placopecten
Pecten
Bivalvia
Capitella (Annelida)
0.2
C
Physa
Aplexa
Physidae
Lymnaea
Radix
Lymnaeidae
Glyptophysa
Ferrissia
Biomphalaria
Planorbis
Planorbidae
Acroloxus Acroloxidae
Latia Latiidae
Chilina Chilinidae
Hygrophila
Siphonaria
Carychium
Laemodonta
Melampus
Limicolaria
Cepaea
Cochlicopa
Arion
Deroceras
Laevicaulis
Aplysia (Opisthobranchia)
0.05
D
E. quaesita (n=7)
E. quaesita (n=6)
E. scaevola
E. murayamai
E. quaesita
E. senckenbergiana (n=3)
E. senckenbergiana
E. senckenbergiana
Mandarina hahajimana
E. peliomphala brandtii
E. sandai
E. decorata
E. decorata
E. decorata
E. grata
E. peliomphala
E. eoa
E. subnimbosa
E. dixoni
E. sp.
E. amaliae
E. callizona
Nesiohelix sp.
0.02
E
P. taeniata (w)
P. suturalis (c)
P. taeniata (w; n=2)
P. tohiveana (w)
P. suturalis(c)
P. tohiveana (c)
P. tohiveana (c)
P. mirabilis (c)
P. taeniata (w)
P. suturalis (c)
P. suturalis (c)
P. taeniata (w)
P. mooreana (c)
P. varia (c)
P. suturalis (c)
P. taeniata (w)
P. suturalis (c)

Figure S2. Related to Figure 1. Molecular phylogenies and networks

(A) Maximum likelihood phylogeny of FH2 protein domain sequences from animals. *Ldia1* and *Ldia2* cluster with diaphanous-related proteins from other taxa, including human and mouse. This classification is further supported by the observation that both *Ldia1* and *Ldia2* genes have a GTPase-binding domain (GBD; interacts with Rho-GTPases), FH3, FH1, and FH2 domains, and a diaphanous autoregulatory domain (DAD; interacts with the GBD domain). Phylogeny based on 319 amino acid positions after gaps removed; LG model, invariant sites (I) and rate variation amongst sites (G), using PhyML 3.1, with bootstrap support shown.

(B). Maximum likelihood phylogeny of FH2 domain DNA sequences from molluscs. *Lymnaea*, *Pomacea*, *Crassostrea* and *Crepidula* all have duplicated formin genes. As in the rRNA phylogeny (C), *Lymnaea* and *Physa* cluster together, however the support for this relationship is low (61% ML bootstraps; *P*=0.96 Bayesian posterior probability). The relatively long branch for *Ldia2* relative to *Ldia1* may reflect the fact that *Ldia2* is probably the derived copy of the gene. Phylogeny based on alignment of ~1280 bp; GTR model, invariant sites (I) and rate variation amongst sites (G), using PhyML 3.1 with bootstrap support shown, alongside Bayesian posterior probability for the same branches. Taxa with substantial missing data are shown with a dotted line.

(C) Maximum likelihood phylogeny showing the relationships, and thus evolution of chirality, within the monophyletic Hygrophilid clade. Dextral and sinistral families show in blue and red, respectively. The phylogeny shows, with good support (97% ML bootstraps; *P*=1 Bayesian posterior probability), that the Lymnaeidae and Physidae are sister groups. Either sinistrality evolved on two separate occasions, or else once with the ancestral Lymnaeid reverting to dextrality. Phylogeny based on concatenated 18S and 28S rRNA sequences; TIM3 model, invariant sites (I) and rate variation amongst sites (G) model, using PhyML 3.1, with bootstrap support >70% shown, alongside Bayesian posterior probability for the same branches. Broken lines indicate some missing data for that taxon.

(D) Maximum likelihood phylogeny showing a lack of association between variation in formin gene intron sequence and chirality in *Euhadra* snails. Phylogeny based on ~640 bp intron sequence from diaphanous-related formin; TPM1uf and rate variation amongst sites (G) model, using PhyML 3.1, with bootstrap support >70% shown, alongside Bayesian support (>0.95) for the same branches. Dextral and sinistral species show in blue and red, respectively.

(E) Network showing relationship showing a lack of association between variation in formin gene intron sequence and chirality in *Partula* snails. NeighbourNet network based on matrix of uncorrected p-distances. Key: dark blue, genetically dextral; light blue, genetically dextral or heterozygote; dark red, genetically sinistral; light red, genetically sinistral or heterozygote; C, captive individual; W, wild individual.

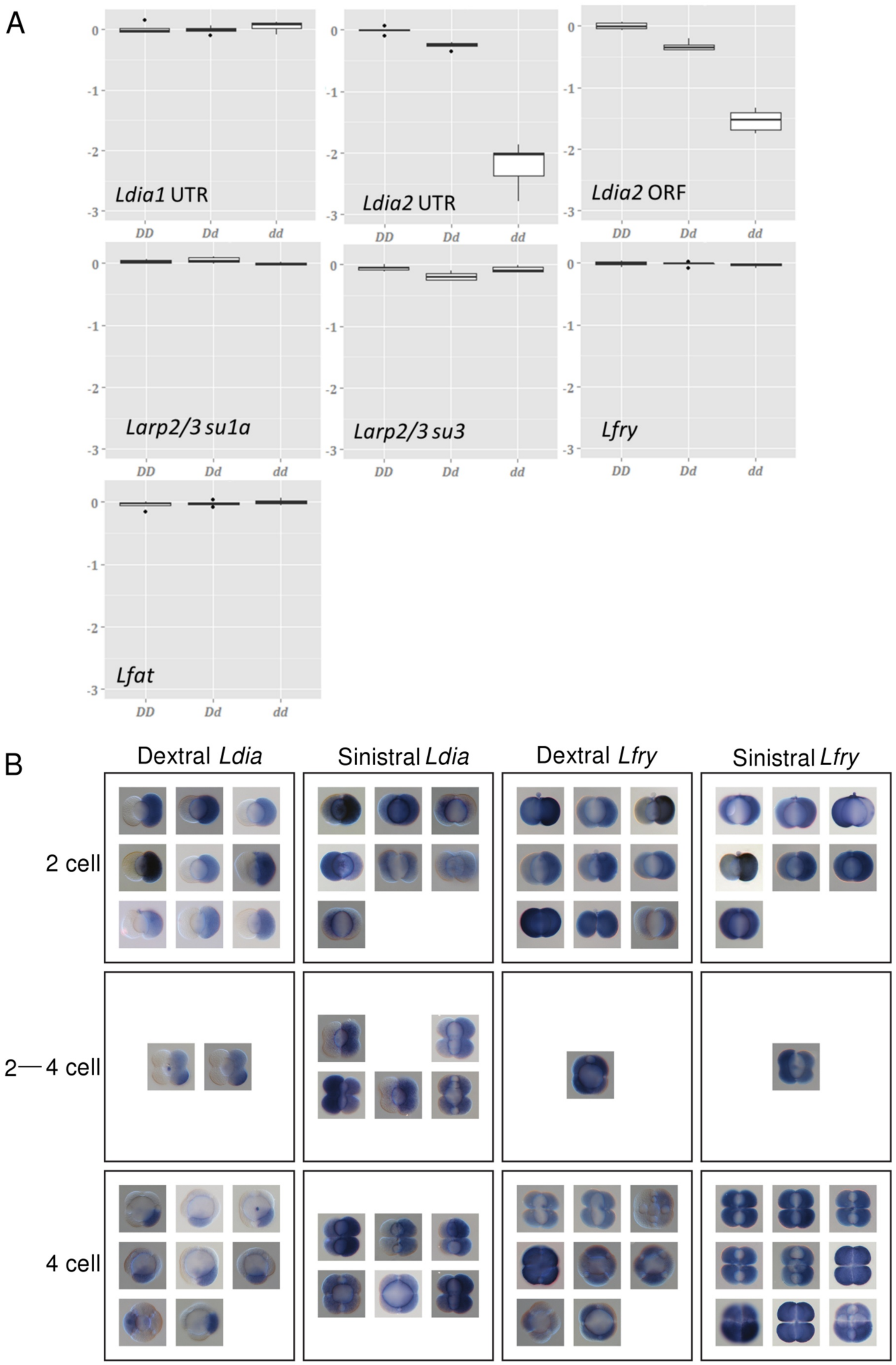

A
Ldia1 UTR
Ldia2 UTR
Ldia2 ORF
Larp2/3 su1a
Larp2/3 su3
Lfry
Lfat
0
-1
-2
-3
DD
Dd
dd
B
Dextral Ldia
Sinistral Ldia
Dextral Lfry
Sinistral Lfry
2 cell
2—4 cell
4 cell

Figure S3. Related to Figure 1; Expression of genes in the early embryo.

(A) Boxplots showing normalised relative quantities (NRQ) of qRT-PCR assays of maternal transcripts in single cell embryos, from dextral homozygote (*DD*), dextral heterozygote (*Dd*) and sinistral recessive homozygote (*dd*) individuals. Significant comparisons were *Ldia2* UTR, *DD* vs *dd* (*P*=0.002), *DD* vs *Dd* (*P*=0.004), *Dd* vs *dd* (*P*=0.004); *Ldia2* ORF, *DD* vs *dd* (*P*=0.002), *DD* vs *Dd* (*P*=0.004), *Dd* vs *dd* (*P*=0.004); *Larp2/3 su1a*, *Dd* vs *dd* (*P*=0.03); *Larp2/3 su3*, *DD* vs *Dd* (*P*=0.009), *Dd* vs *dd* (*P*=0.030). Values relative to a DD calibrator sample and normalised to three endogenous control genes. No significant differences in expression were detected for *Lmfsd*, *Lprcp* and *Ltud* (not shown).

(B) Whole mount *in situ* images, showing expression of genes in dextral and sinistral *L. stagnalis* embryos. In dextral embryos, *Ldia* showed strongly asymmetric in expression in both 2 cell and 4 cell embryos. In comparison, expression of *Ldia* in sinistral embryos showed more generalised, less obviously asymmetric staining and considerably more variation between individuals. In dextrals and sinistrals, *Lfry* sometimes showed an asymmetric pattern, albeit less striking when compared with *Ldia*, and with more variation between individuals. Note: due to high sequence identity, the formin probe was reactive against both *Ldia1* and *Ldia2*.

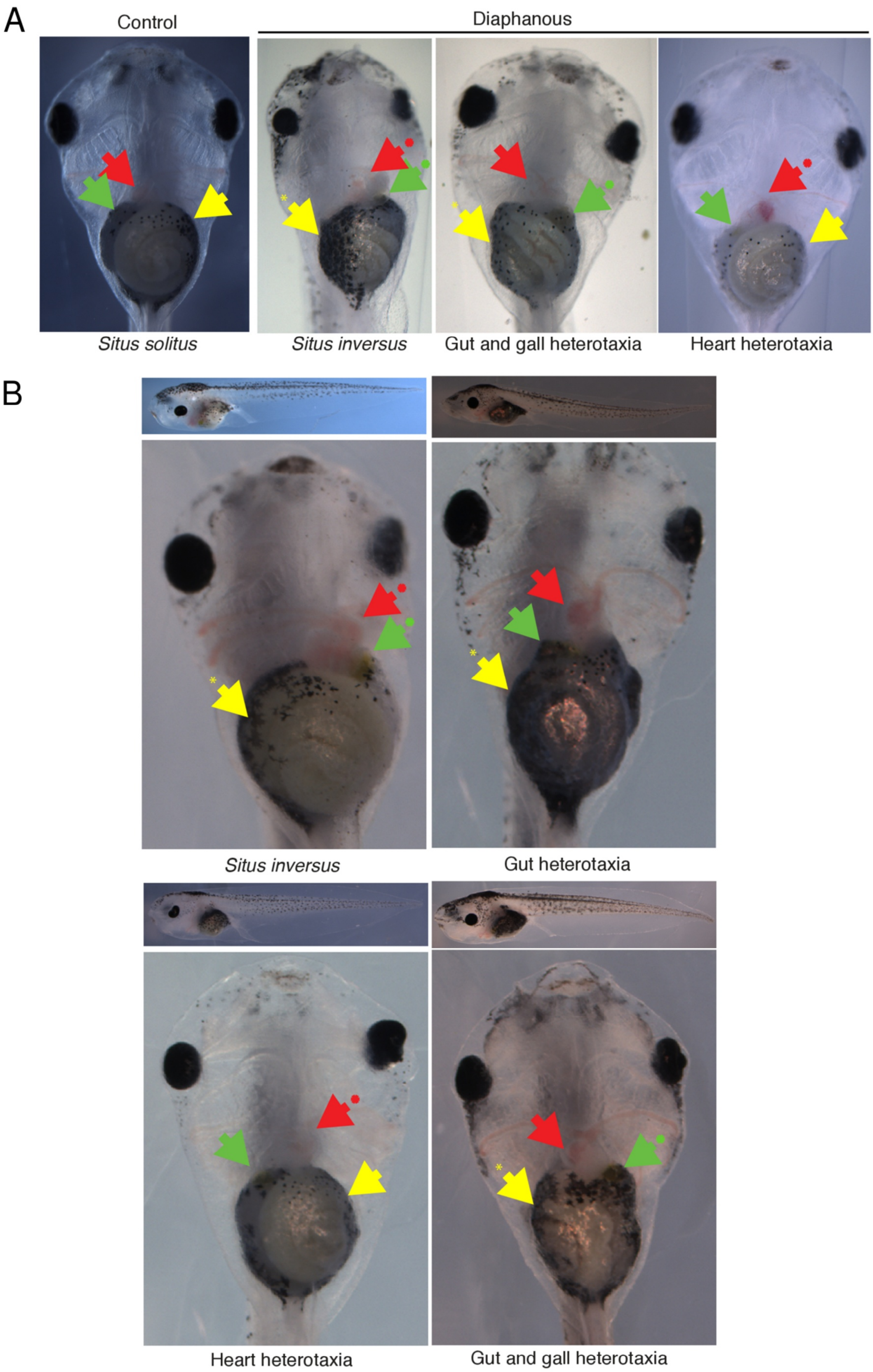
A
Control
Diaphanous
Situs solitus
Situs inversus
Gut and gall heterotaxia
Heart heterotaxia
B
Situs inversus
Gut heterotaxia
Heart heterotaxia
Gut and gall heterotaxia

Figure S4. Related to Figure 4. Organ *situs* for treated *Xenopus* tadpoles at Stage 45

(A) Examples of organ *situs* for experimental microinjection with wild type mouse *dia1* mRNA. The control shows a wild-type (*situs solitus*) tadpole, ventral view, demonstrating the normal arrangement of the stomach (yellow arrowhead), heart apex (red arrowhead), and gall bladder (green arrowhead). Heterotaxic tadpoles (ventral view) resulting from diaphanous overexpression show reversal of all three organs, i.e. *situs inversus*, the gut position and looping and gall bladder, or just the heart.

(B) Examples of organ *situs* for experimental treatment with CK-666. Full length images of tadpoles demonstrate that tadpoles look otherwise normal in terms of correct dorsal-ventral axis formation.

	Snail ID		rad7	rad4	b1	b2	b3	b4	b5	b6	b7	b8	b9	b10	b11	b12	b13	b14	b15	b16	rad5
			**Genotypes of anonymous markers***																		
**(A)**	**SINISTRALS**	**Chirality genotype (inferred from offspring)**									**Non-recombined haplotype**										
	1S6	*dd*	*bb*	*bb*	*bb*	*bb*	*bb*	*bb*	*bb*	*bb*		*bb*	*bb*	*bb*	*bb*		*bb*	*Bb*	*Bb*	*Bb*	*Bb*
	**245**	*dd*	***Bb***	***Bb***	***Bb***	***Bb***	***Bb***	***Bb***	***Bb***	***Bb***	***bb***	***bb***	***bb***	***bb***			***bb***		***bb***	***bb***	***bb***
	270	*dd*	*bb*	*bb*	*bb*	*bb*	*bb*	*bb*	*bb*			*bb*	*bb*	*bb*	*bb*		*bb*	*Bb*	*Bb*	*Bb*	*Bb*
	291	*dd*	*bb*	*bb*	*bb*	*bb*	*bb*	*bb*	*bb*			*bb*	*bb*	*bb*		*bb*	*bb*	*bb*	*bb*	*bb*	*Bb*
	303	*dd*	*bb*	*bb*	*bb*	*bb*	*bb*	*bb*	*bb*		*bb*	*bb*	*bb*	*bb*	*bb*		*bb*		*Bb*	*Bb*	*Bb*
	310	*dd*	*bb*	*bb*	*bb*	*bb*	*bb*	*bb*	*bb*			*bb*	*bb*	*bb*	*bb*	*bb*	*bb*	*Bb*	*Bb*	*Bb*	*Bb*
	338	*dd*	*bb*	*bb*	*bb*	*bb*	*bb*	*bb*	*bb*			*bb*	*bb*	*bb*	*bb*		*bb*	*Bb*	*Bb*	*Bb*	*Bb*
	**397**	*dd*	***bb***	***bb***	***bb***	***bb***	***bb***	***bb***	***bb***			***bb***	***bb***	***bb***	***bb***	***Bb***	***Bb***	***Bb***	***Bb***	***Bb***	***Bb***
	472	*dd*	*bb*	*bb*	*bb*	*bb*	*bb*	*bb*	*bb*			*bb*	*bb*	*bb*	*bb*		*bb*	*Bb*	*Bb*	*Bb*	*Bb*
	514	*dd*	*bb*	*bb*	*bb*	*bb*	*bb*	*bb*	*bb*			*bb*	*bb*	*bb*	*bb*		*bb*	*Bb*	*Bb*	*Bb*	*Bb*
	583	*dd*	*bb*	*bb*	*bb*	*bb*	*bb*	*bb*	*bb*			*bb*	*bb*	*bb*	*bb*		*bb*	*Bb*	*Bb*	*Bb*	*Bb*
	602	*dd*	*bb*	*bb*	*bb*	*bb*	*bb*	*bb*	*bb*			*bb*	*bb*	*bb*	*bb*		*bb*	*Bb*	*Bb*	*Bb*	*Bb*
	618	*dd*	*bb*	*bb*	*bb*	*bb*	*bb*	*bb*	*bb*			*bb*	*bb*	*bb*			*bb*	*bb*	*Bb*	*Bb*	*Bb*
	650	*dd*	*bb*	*bb*	*bb*	*bb*	*bb*	*bb*	*bb*			*bb*	*bb*	*bb*			*bb*	*bb*	*Bb*	*Bb*	*Bb*
	815	*dd*	*bb*	*bb*	*bb*	*bb*	*bb*	*bb*	*bb*			*bb*	*bb*	*bb*	*bb*	*bb*	*bb*	*Bb*	*Bb*	*Bb*	*Bb*
	958	*dd*	*bb*	*bb*	*bb*	*bb*	*bb*	*bb*	*bb*			*bb*	*bb*	*bb*	*bb*		*bb*	*Bb*		*Bb*	*Bb*
	**982**	*dd*	***Bb***	***Bb***	***Bb***	***Bb***	***Bb***	***bb***	***bb***			***bb***	***bb***	***bb***			***bb***	***bb***	***bb***	***bb***	***bb***
	989	*dd*	*bb*	*bb*	*bb*	*bb*	*bb*	*bb*	*bb*			*bb*	*bb*	*bb*			*bb*	*bb*		*Bb*	*Bb*
	1016	*dd*	*bb*	*bb*	*bb*	*bb*	*bb*	*bb*	*bb*			*bb*	*bb*	*bb*	*bb*		*bb*	*Bb*	*Bb*	*Bb*	*Bb*
	1021	*dd*	*bb*	*bb*	*bb*	*bb*	*bb*	*bb*	*bb*			*bb*	*bb*	*bb*	*bb*		*bb*	*Bb*		*Bb*	*Bb*
	1351	*dd*	*bb*	*bb*	*bb*	*bb*	*bb*	*bb*	*bb*			*bb*	*bb*	*bb*	*bb*		*bb*	*bb*	*Bb*	*Bb*	*Bb*
	1726	*dd*	*bb*	*bb*	*bb*	*bb*	*bb*	*bb*	*bb*			*bb*	*bb*	*bb*	*bb*		*bb*	*Bb*	*Bb*	*Bb*	*Bb*
	1740	*dd*	*bb*	*bb*	*bb*	*bb*	*bb*	*bb*	*bb*			*bb*	*bb*	*bb*	*bb*		*bb*	*Bb*	*Bb*	*Bb*	*Bb*
	2021	*dd*	*bb*	*bb*	*bb*	*bb*	*bb*	*bb*	*bb*			*bb*	*bb*	*bb*	*bb*		*bb*	*Bb*	*Bb*	*Bb*	*Bb*
	2302	*dd*	*bb*	*bb*	*bb*	*bb*	*bb*	*bb*	*bb*			*bb*	*bb*	*bb*	*bb*		*bb*		*Bb*	*Bb*	*Bb*
	2352	*dd*	*bb*	*bb*	*bb*	*bb*	*bb*	*bb*	*bb*			*bb*	*bb*	*bb*	*bb*		*bb*	*Bb*	*Bb*	*Bb*	*Bb*
	2371	*dd*	*bb*	*bb*	*bb*	*bb*	*bb*	*bb*	*bb*			*bb*	*bb*	*bb*	*bb*		*bb*		*Bb*	*Bb*	*Bb*
	2548	*dd*	*bb*	*bb*	*bb*	*bb*	*bb*	*bb*	*bb*			*bb*	*bb*	*bb*	*bb*		*bb*	*Bb*	*Bb*	*Bb*	*Bb*
	2561	*dd*	*bb*	*bb*	*bb*	*bb*	*bb*	*bb*	*bb*			*bb*	*bb*	*bb*			*bb*	*bb*	*bb*	*bb*	*Bb*
	2592	*dd*	*bb*	*bb*	*bb*	*bb*	*bb*	*bb*	*bb*			*bb*	*bb*	*bb*	*bb*		*bb*	*Bb*	*Bb*	*Bb*	*Bb*
	2595	*dd*	*bb*	*bb*	*bb*	*bb*		*bb*	*bb*			*bb*	*bb*	*bb*	*bb*		*bb*	*Bb*	*Bb*	*Bb*	*Bb*
	**2597**	*dd*	***Bb***	***Bb***	***bb***	***bb***	***bb***	***bb***	***bb***		***bb***	***bb***	***bb***	***bb***			***bb***	***bb***	***bb***	***bb***	***bb***
	3046	*dd*	*bb*	*bb*								*bb*	*bb*	*bb*	*bb*		*bb*				*Bb*
	3216	*dd*	*bb*	*bb*										*bb*	*bb*		*bb*				*Bb*
	3349	*dd*	*bb*	*bb*										*bb*	*bb*		*bb*				*Bb*
	3358	*dd*	*bb*	*bb*										*bb*	*bb*		*bb*				*Bb*
	**3465**	*dd*	***bb***	***bb***										***bb***	***bb***	***bb***	***Bb***				***Bb***
	3535	*dd*	*bb*	*bb*										*bb*	*bb*		*bb*				*Bb*
	3536	*dd*	*bb*	*bb*										*bb*	*bb*		*bb*				*Bb*
	3538	*dd*	*bb*	*bb*										*bb*	*bb*		*bb*				*Bb*
	3541	*dd*	*bb*	*bb*	*bb*					*bb*	*bb*	*bb*		*bb*	*bb*		*bb*				*Bb*
	3563	*dd*	*bb*	*bb*										*bb*	*bb*		*bb*				*Bb*
	3579	*dd*	*bb*	*bb*										*bb*	*bb*		*bb*				*Bb*
**(B)**	**DEXTRALS**	**Chirality genotype (inferred from offspring and genotype)**																			
	669	*Dd*	*Bb*	*Bb*	*Bb*		*Bb*	*Bb*	*Bb*	*Bb*	*Bb*	*Bb*		*Bb*	*Bb*		*Bb*	*Bb*	*bb*	*bb*	*bb*
	768	*Dd*	*Bb*	*Bb*			*Bb*	*Bb*	*Bb*		*Bb*	*Bb*	*Bb*	*Bb*	*Bb*		*Bb*	*bb*	*bb*	*bb*	*bb*
	843	*Dd*	*Bb*	*Bb*	*Bb*		*Bb*	*Bb*	*Bb*		*Bb*	*Bb*		*Bb*	*Bb*		*Bb*	*Bb*	*bb*	*bb*	*bb*
	**1209**	*Dd*	***Bb***	***Bb***	***Bb***		***Bb***	***Bb***	***Bb***		***Bb***	***Bb***	***Bb***	***Bb***	***Bb***	***bb***	***bb***	***bb***	***bb***	***bb***	***bb***
	1282	*Dd*	*Bb*	*Bb*	*Bb*		*Bb*	*Bb*	*Bb*			*Bb*	*Bb*	*Bb*	*Bb*		*Bb*	*bb*	*bb*	*bb*	*bb*
	1902	*Dd*	*Bb*	*Bb*	*Bb*		*Bb*	*Bb*	*Bb*		*Bb*	*Bb*	*Bb*	*Bb*	*Bb*		*Bb*	*Bb*	*Bb*	*Bb*	*bb*
	1910	*Dd*	*Bb*	*Bb*	*Bb*		*Bb*	*Bb*	*Bb*		*Bb*	*Bb*	*Bb*	*Bb*	*Bb*		*Bb*	*bb*	*bb*	*bb*	*bb*
	1931	*Dd*	*Bb*	*Bb*	*Bb*		*Bb*	*Bb*	*Bb*		*Bb*	*Bb*	*Bb*	*Bb*	*Bb*		*Bb*	*bb*	*bb*	*bb*	*bb*
	2236	*Dd*	*Bb*	*Bb*	*Bb*		*Bb*	*Bb*	*Bb*			*Bb*		*Bb*	*Bb*		*Bb*	*Bb*	*Bb*	*Bb*	*bb*
	2804	*Dd*	*Bb*	*Bb*			*Bb*	*Bb*	*Bb*			*Bb*	*Bb*	*Bb*	*Bb*		*Bb*	*bb*	*bb*	*bb*	*bb*
	2827	*Dd*	*Bb*	*Bb*			*Bb*	*Bb*	*Bb*			*Bb*	*Bb*	*Bb*	*Bb*		*Bb*	*bb*	*bb*	*bb*	*bb*
	2996	*Dd*	*Bb*	*Bb*								*Bb*	*Bb*	*Bb*	*Bb*	*Bb*	*Bb*				*bb*
	3211	*Dd*	*Bb*	*Bb*	*Bb*					*Bb*	*Bb*	*Bb*		*Bb*	*Bb*		*Bb*				*bb*
	3399	*Dd*	*Bb*	*Bb*										*Bb*	*Bb*		*Bb*				*bb*
**(C)**	**DEXTRALS (non-informative)**	**Chirality genotype (inferred from offspring and genotype)**																			
	3402	*DD*	*BB*	*BB*										*BB*			*BB*				*Bb*
	3D8	*DD*	*BB*	*BB*	*BB*		*BB*	*BB*	*BB*	*BB*		*BB*	*BB*	*BB*			*BB*	*Bb*	*Bb*	*Bb*	*Bb*
	757	*Dd*	*Bb*	*Bb*	*Bb*		*Bb*	*Bb*	*Bb*	*Bb*		*Bb*		*Bb*			*Bb*	*Bb*	*Bb*	*Bb*	*BB*
	840	*Dd*	*Bb*	*Bb*	*Bb*		*Bb*	*Bb*	*Bb*			*Bb*		*Bb*			*Bb*	*BB*	*BB*	*BB*	*BB*
	1053	*DD*	*BB*	*BB*	*BB*		*BB*	*BB*	*BB*	*BB*		*BB*		*BB*			*BB*	*Bb*	*Bb*	*Bb*	*Bb*
	1148	*DD*	*BB*	*BB*	*BB*			*BB*	*BB*			*BB*		*BB*			*BB*	*Bb*	*Bb*	*Bb*	*Bb*
	1220	*DD*	*BB*	*BB*	*BB*		*BB*	*BB*	*BB*	*BB*	*BB*	*BB*		*BB*			*BB*	*Bb*	*Bb*	*Bb*	*Bb*
	1235	*Dd*	*Bb*	*Bb*	*Bb*		*Bb*	*Bb*	*Bb*			*Bb*		*Bb*			*Bb*	*BB*	*BB*	*BB*	*BB*
	1793†	*DD/Dd*	*BB*	*BB*	*BB*		*BB*	*BB*	*BB*		*BB*	*BB*		*BB*			*Bb*	*Bb*	*Bb*	*Bb*	*Bb*
	1967	*Dd*	*BB*	*BB*	*BB*		*BB*	*BB*	*BB*	*BB*	*Bb*	*Bb*		*Bb*			*Bb*	*Bb*	*Bb*	*Bb*	*Bb*
	1970	*DD*	*BB*	*BB*	*BB*		*Bb*	*BB*	*BB*			*BB*		*BB*			*BB*	*Bb*	*Bb*	*Bb*	*Bb*
	2104	*Dd*	*Bb*	*Bb*	*Bb*		*Bb*	*Bb*	*Bb*			*Bb*		*Bb*			*Bb*	*B*	*BB*	*BB*	*BB*
	2179	*Dd*	*Bb*	*Bb*	*Bb*		*Bb*	*Bb*	*Bb*								*Bb*	*B*	*BB*	*BB*	*BB*
	2222†	*DD/Dd*	*BB*	*BB*	*BB*		*B*	*BB*												*Bb*	*Bb*
	2270	*DD*	*BB*	*BB*	*BB*		*BB*	*BB*	*BB*			*BB*		*BB*				*BB*	*Bb*	*Bb*	*Bb*
	2407	*Dd*	*Bb*	*Bb*	*Bb*		*Bb*	*Bb*	*Bb*			*Bb*		*Bb*			*Bb*	*BB*	*BB*	*BB*	*BB*
	2446	*Dd*	*Bb*	*Bb*	*Bb*		*Bb*	*Bb*	*Bb*			*Bb*		*Bb*			*Bb*	*BB*	*BB*	*BB*	*BB*
	2784	*Dd*	*Bb*	*Bb*			*Bb*	*Bb*	*b*			*Bb*		*Bb*			*Bb*		*BB*	*BB*	*BB*
	2797	*DD*	*BB*	*BB*			*BB*	*BB*	*B*		*BB*	*BB*		*BB*			*BB*	*Bb*	*Bb*	*Bb*	*Bb*
	2830	*Dd*	*Bb*	*Bb*				*Bb*	*Bb*			*Bb*		*Bb*			*Bb*	*BB*	*BB*	*BB*	*BB*
	2862	*Dd*	*Bb*	*Bb*								*Bb*		*Bb*			*Bb*				*BB*
	2906	*DD*	*BB*	*BB*							*BB*	*BB*		*BB*			*BB*				*Bb*
	2992	*DD*	*BB*	*BB*							*BB*	*BB*		*BB*		*BB*	*BB*				*Bb*
	2999	*DD*	*Bb*	*Bb*	*BB*		*BB*			*BB*	*BB*	*BB*		*BB*		*BB*	*BB*				*BB*
	3150	*Dd*	*Bb*	*Bb*								*Bb*		*Bb*		*Bb*	*Bb*				*BB*

Table S1. Related to Figure 1. Individuals used to fine map the chirality locus, *D*, and their genotypes. Anonymous tags are shown according to their physical order on the snail chromosome; genotype name is arbitrary, but case corresponds to the same as chirality genotype. Three types of snail were used in the mapping:

(A) Sinistral snails were always informative because the chirality genotype (*dd*) is certain, and so the relative position can be mapped.

(B) Only some dextrals were informative, specifically those individuals which inherited a sinistral-derived chromosome and another that was recombinant between rad4 and rad5. They were useful (especially snail 1209) because the chirality gene *D* must be outwith the region that is otherwise homozygote.

(C) The remaining dextrals are not useful for mapping, because it is unknown if their chirality genotype is *DD* or *Dd*.

† indicates individual not included in count for Figure 1, because breakpoint not finely mapped. Dotted line indicates extent of non-recombined haplotype (must contain the chirality mutation). Grey shading indicates location of putative recombination break point.

	SMIFH2 (100 µM)						CK-666 (100 µM)					
Time of treatment (mins)	**0**	**10**	**30**	**45**	**60**	**ctrl**	**0**	**10**	**30**	**45**	**60**	**ctrl**
Number of embryos	274	322	170	111	76	219	196	155	147	131	113	190
Number of treatments	7	6	3	3	3	11	6	7	6	5	5	5
Number treatments ≥5 embryos developed to 8 cell	6	6	3	3	3	11	3	4	5	5	5	5
**Across treatments:**												
Percent developed to 8 cell	26.1	65.6	72.5	84.6	88.0	95.3	32.5	46.1	61.1	100	99.2	99.4
S.E.	12.6	12.7	22.8	15.4	12.0	2.6	16.7	18.1	14.4	0	0.8	0.6
Mean angle of rotation	14.1	19.5	27.0	35.8	39.4	37.1	20.5	26.0	27.3	31.0	31.9	32.7
S.E.	2.5	1.6	1.3	2.2	1.0	1.3	1.9	1.1	0.8	1.9	3.3	2.1
Percent neutral embryos	28.9	19.5	4.8	0	0	0	7.4	0	1.2	0	2.7	0
S.E.	7.0	3.7	2.2	0	0	0	5.7	0	1.2	0	1.9	0
Percent neutral micromeres	43.4	41.5	19.8	2.8	1.0	1.4	21.6	8.8	11.6	8.1	5.8	4.4
S.E.	4.4	3.3	3.0	1.4	0.5	0.5	6.0	3.3	3.2	2.1	2.6	1.1

Table S2. Related to Figure 2. Effect of inhibitors on development of embryos to 8-cell stage.

Treatment	Uninjected	DMSO			CK666 25 µM			50 µM			SMIFH2 25 µM			50 µM		
NF Stages	All	1-6	8-14	19-21	1-6	8-14	19-21	1-6	8-14	19-21	1-6	8-14	19-21	1-6	8-14	19-21
% heterotaxia	1	2	0	3	0	2	17	7	3	16	4	0	0	13	4	12
N	755	354	333	312	64	126	109	205	413	355	71	55	108	345	277	128
X^2 for randomization	1	0.132	0.233	< 0.001	0.439	0.439	< 0.001	< 0.001	< 0.001	< 0.001	0.111	0.323	0.166	< 0.001	0.018	< 0.001

Table S3. Related to Figure 4. Organ heterotaxia for frog embryo treatments.

NF Stages refers to developmental stages identified in the Normal Table of *Xenopus* development [S1]. X^2 for randomization is calculated relative to untreated control embryos.

Supplemental Experimental Procedures

Source of animals, crosses and husbandry

Although the snail family Lymnaeidae is predominantly dextral, sinistrality has been observed in four species [S2, S3, S4, S5]. The first experiments on chirality in *Lymnaea* were originally carried out using sinistral *L. peregra* [S2], but this strain is no longer in culture. Recent work [S6, S7] has therefore all been carried out using sinistral *L. stagnalis*, which all ultimately derive from the same source [S5]. In our experiments, dextral *L. stagnalis* were originally removed from a pond within the University Park, University of Nottingham, with snails being maintained as previously described [S8]. For the majority of the experiments (e.g. qRT-PCR; drug treatments), we used near-isogenic lines (>99%) of sinistrals and dextrals, created by repeated backcrossing [S9], with the exception of heterozygote snails used in the qRT-PCR experiments, which were hybrids produced by crossing the near-isogenic lines (phenotypically sinistral snails that produce dextral offspring are genetically *Dd*).

We also used two other chirally variable snail genera, to test associations between candidate genes and chirality. Land snails of the genus *Euhadra* vary both within and between species. Although the genetics of chirality is not known, because *Euhadra* of opposite chirality are unable to mate [S10], we were able to infer chiral genotype from the geographic distribution of the different species and populations. *Partula* are another chirally variable genus, originally found across Polynesia but now endangered. We used frozen material of this genus, as well as specimens donated by Edinburgh Zoo. In *P. suturalis*, sinistral is dominant to dextral [S11].

For *Xenopus laevis*, this study was carried out in strict accordance with the recommendations in the Guide for the Care and Use of Laboratory Animals of the National Institutes of Health. The protocol was approved by Tufts University's Institutional Animal Care and Use Committee (Tufts IACUC protocol #M2014-79). *Xenopus* embryos were collected and maintained according to standard protocols [S12] in 0.1 x Modified Marc's Ringers (MMR), pH 7.8, and staged according to Nieuwkoop and Faber [S1].

BAC library screening, BAC walking and fluorescent *in situ* hybridisation

As previously described [S9], a ~10-fold coverage BAC library was constructed in the vector pIndigoBAC-5 by Bio S&T Inc. (Montreal, Canada), using as starting material a mixture of *DD*, *Dd* and *dd* snails. To isolate BACs, PCR amplicons were designed and tested in the laboratory, then were used by Bio S&T Inc for bulk PCR identification of clones. Inserts were isolated from BAC DNA by digestion with *Not*I and sized using pulse field gel electrophoresis. Large scale DNA preparations of positive clones were then prepared using the QIAGEN Large Construct kit according to the manufacturer's instructions. BAC ends were sequenced using BigDye version 3.1 and the T7 primer TAATACGACTCACTATAGGG (FP1 end) and pIndigoBAC-5 TACGCCAAGCTATTTAGGTGAGA (RP2 end). Further information on the FISH method was described previously [S9].

In the first round of the BAC walk (#1) pairs of BACs were isolated corresponding to RAD-seq derived markers *rad4* (BACs red6F, red8H), rad5 (blue2D, blue10A) and rad7 (green1A, green12D) (three of these clones were described previously [S9]). Thereafter, in an attempt to minimise the number of steps, we walked from locus *rad4*, because we detected relatively few recombinants between *rad4* and *D*, compared with between *rad5* and *D*. PCR amplicons were designed from each BAC-end of red6F and red8H sequence and the non-overlapping ends identified. As the orientation of non-overlapping BAC ends was not known, the next walk (#2) was conducted from both extreme ends (red6F RP2, red8H FP1) as well as including a further internal amplicon (red6F FP1). From this, six further BAC clones were identified: from red6F RP2 (green4A and green5F); from red8H FP1 (red3E and red11F); from red6f FP1 (blue8G, blue12F).

To inform the direction of the BAC walk, and the approximate number of steps that might be necessary to cover the region, fibre-FISH was carried out using adult snail ovotestis, liver tissue and albumen gland as a source of chromosomes, as previously described in detail [S9]. Although few intact fibre-FISH images were obtained, most likely because of the relatively great distance between the BACs, the gap between *rad4* and *rad5* was estimated to be ~0.4 Mb; the direction of the walk was informed by a 3-colour image, using green4A/red6F and red3E/red6F (all on or adjacent to rad4) versus blue2D (over *rad5*) (Figure S1). This was also corroborated by identifying a single recombination break-point between the *rad4* marker and red3E RP2 (Table S1). This showed that red3E RP2 was the most appropriate BAC end for the next walk.

Subsequent rounds of BAC walking were made considerably simpler because of the known orientation. Green12H/red10B were isolated (#3), then green4F/red9G (#4), yellow10G/white9F (#5), yellow2B/blue3G (#6), green11A (#7), white7E/yellow5F (#8), blue10B/red7F (#9), and white10A/red3A (#10). We also attempted to walk from rad5, recovering BACs yellow3C/red9F (#11) and yellow11H/white8C (#12).

Despite the apparent proximity of the marker rad4 to *D*, as inferred by recombination, we did not bridge the gap between *D* and *rad5*. Fortunately, bridging the gap was ultimately unnecessary, as the ten walks from rad4 spanned the whole of the non-recombined haplotype, and thus the region that must contain the mutation (Table S1; physically, *D* is closer to *rad5* than *rad4*, but separated by a warm spot of recombination and an unbridged gap).

Fine mapping

Previously, we used a large cross to identify offspring that showed evidence of recombination between rad4 and rad5 [S9]. These individuals were used to map recombination break points across the *D*-containing region, and ultimately, to identify the non-recombined haplotype that must contain the mutation. The majority of markers used were derived from BAC-end Sanger sequences. First, for each BAC end, sequence polymorphism between the sinistral and dextral haplotypes was identified. Then, by designing an appropriate assay using SNPS2CAPS [S13] or using a convenient length polymorphism, we screened the potential recombinant individuals for up to 16 markers between rad4 and rad5. Of these markers, the most informative in terms of mapping recombination breakpoints (shown in Figure 1) were b1 (F: GGGAGATTAAACACGCGGTA; R: AAAAAGGGGCTCGGTCTAAA; 635 bp product; *Btg*I digest), b3 (F: ATGGCAAAAGGACAACAACC; R: TTCTCTTGGCACAGTCAACG; 460 bp; indel), b6 (F: GATTTCATATTGCGCGTGAG; R: AAAAGGTTGCGTGAAACTGC; 420 bp; *Nla*III), b7 (F: TTGCTGCCCTTATTAAGTTTCC; R: CTGGTCACAAGTGAGCAATTC; 407 bp; indel), b8 (F: YGGRCCAACATTTATTTYCGTTAC; R: GTCATGGAMATGGTGCAGAG; 310 bp; indel), b11 (F: TTCCTTGGCTTCTTTTGCTC; R: TGTTTCCAGCAATCCTTTTG; 305 bp; reverse primer sequence), b12 (F: GTGTCCTCATTTTCCMATCC; R: AGYCACGTAGGCTGTGAAAC; 170 bp; *Stu*I), b13 (F: TTATGTTCATAAAGCGCATACAAG; R: CAATTTCAACTTTGTGAATTGGAG; 440 bp; *Xba*I). Eight other markers were also used: b2 (F: CCAAATTTAAAACCGTCTCATCC; R: CCACCCAAACACCCATACAG; 401 bp; *Bam*HI), b4 (F: ACCAAATGCTGGGAAACAAG; R: GGTCCAGTACATGCAACACG; 454 bp; reverse primer sequence), b5 (F: CCCGTGAAAACTAAAACGATG; R: ATAGGTGCGAGGCRCTTTTG; 684 bp; *Bst*EII), b9 (F: ACTTAATCAGAAACATCCGTGTC; R: ACGTGCCATGTGATTTTAGC; 430 bp; *Hin*dIII), b10 (F: GGCATATTTAGATGCATAGTCAAGG; R: CATCAGTCATGCCAGGTATTG; 402 bp; *Bsp*1286I), b14 (F: AACCGGAACCACAGACTGAC; R: CGTCCACATCGGACGTAAC; 607 bp; *Dde*I), b15 (F: TGACTGTCAAGCCACCATTG; R: GCTGCAGTAGGATGCTAACAAG; 413 bp; reverse primer sequence), and b16 (F: AATTTAATTGGAGTGAATAATTGAGTG; R: AACCTTTGTTAACTTACYGGAATCG; 370 bp; *Hpy*CH4IV).

Genome and BAC sequencing, assembly and variant calling

Two paired-end (200 and 400 bases insert size; 165,943,096 and 101,468,245 base pairs) and two mate-pair libraries (2,800 and 4,500 bases insert size; 66,964,771 and 71,182,903 base pairs) were generated with Illumina sequencing. Adapters were removed with Cutadapt [S14] and low quality bases were trimmed with Trimmomatic [S15]. The trimmed reads were error corrected with Quake [S16]. Reads were assembled using SOAPdenovo2 [S17] with a kmer size of 61 bases. Gaps were filled post-assembly with Gapcloser [S17]. The genome was assembled in 997,095,996 bases in 148,229 scaffolds with an N50 of 44,738 bases.

To assemble BAC sequences, paired-reads were merged with PEAR [S18]. Adapters were removed with Cutadapt and low quality bases were trimmed with Trimmomatic. Reads were mapped to *E. coli* and vector with Bowtie2 [S19] and removed. BACs with high read coverage were subsampled so that each BAC will have almost the same read coverage. All reads were pooled together and were assembled with SPAdes [S20] using also the mate pair information from whole genome sequencing libraries. Low coverage scaffolds were identified as mis-assemblies and were removed.

For the BAC annotation, the MAKER2 [S21] predictions were used to train Augustus [S22] and the species-specific generated config files were used in Augustus to predict genes in the BAC regions. Genes with BLAST similarity to NCBI nr database or with Pfam domains were retained. To call BAC variants, the four libraries from whole genome sequencing and all the reads from the BACs were mapped to the BACs assembly with Bowtie2. Samtools package [S23] was used to remove duplicates and index the BAM file. Variants were detected using Freebayes [S24]. Protein-coding genes were predicted in the region of interest using the config files from Augustus which were generated for *L. stagnalis* during genome annotation. In addition, the formin genes were identified in the region, by using Exonerate [S25] to align the transcript to the scaffold. In addition to formin, four other genes were identified in the same region and functional annotation was attributed based on similarity to known proteins using BLAST. Bedtools [S26] package was used to identify polymorphisms in exonic regions and each polymorphism was visually inspected in Tablet [S27] for confirmation.

cDNA synthesis

We used the Clontech SMARTer RACE 5'/3' and Advantage 2 PCR kits to obtain the full-length or near full-length sequence of RNA transcripts of both *Ldia1* and *Ldia2* (Genbank KU341302- KU341305), following the protocol according to the manufacturer's instructions. As the sequences *Ldia1* and *Ldia2* are similar, we tried to design gene-specific primers. To clone the UTRs, the primers used for *Ldia1* were candA-N (GAGTGGAGCCTTCTTTTCTTCAGTGAGG) for the 5' UTR and candA-C (AGAGAGATGGTGGGAAAAGACGTACGCC) for the 3' UTR. For *Ldia2*, the primers were paraB-N (TCCATCCTTTGATCTGAGCGGTGCCTTCT) for the 5' UTR and paraB-C (AGGCGAACACCCAGGGCTGCAGGAGAC) for the 3' UTR. As well as the standard M13F and M13R primers, a series of internal primers were also necessary to sequence the full length cDNAs, including Ldia_int1 (ACCACACACTGTTAAACCTTCC), Ldia_int2 (CAGTCAGTGCGTTGTCTAGC), Ldia_int3 TGAGGCTGTTCGTTTGTTGG, Ldia_int4 (TTGAAGACCCTGGAGAGATC), Ldia_int5 (TGTTGTCCTGACTCTTTGTC), Ldia_int6 (GGGTCATCCAATGTTGCC), Ldia_int7 (CAGTTGAGTCAACTCCAGCTC), For_Uni_F and For_UniR1b (both below). For Sanger sequencing, these primers worked on both *Ldia1* and *Ldia2* amplicons. The cDNAs for *Ldia1* and *Ldia2* were ~4.6 kb and 6.4 kb respectively, of which ~3.2 kb is coding sequence.

To investigate putative associations between diaphanous-related formins and chirality in other snail species, we first mined the databases to produce an alignment of mollusc *dia* gene sequences, also including annelid (*Capitella telata*) and human sequences (*Homo sapiens*). Degenerate primers were designed in the conserved FH2 domain region, where the forward primer For_Uni_F was TGGHTCCAGAAAYGCACAGTC and the reverse was For_Uni_R1b TAAACGCYGARCCAGTYTTY, using an internal primer For_Uni_R1a CYGARCCAGTYTTYARAGCTTC for a second round of PCR, followed by Sanger sequencing. Approximately 670 bp of the *dia* cDNA was isolated from *Biomphalaria glabrata*, *Physa acuta*, *Euhadra senckenbergiana* and *Partula mooreana* (Genbank KU341298- KU341301).

Based on the initial derived sequences (above), we subsequently designed species specific intron-spanning primers. For the genus *Euhadra*, Euhfor_F1 GTTGGCTTTGATGTCAGCTTC and Euhfor_R1 AGCTGCCTTATCCACATGG amplified ~170 bp of *dia* coding sequence plus an intron of variable size, ~600 bp in most, but ~1.6 kb for *E. grata* (Genbank KU341262-KU341297). For the genus *Partula*, Parfor_F1 ATCGGTTTTGACATCAGCTTTT and Parfor_R1 AGCAGCCTTATCCACATGAAT amplified ~800 bp fragment, including ~70 bp of *dia* coding sequence and an intron (Genbank KU341240-KU341261).

Candidate genes

Recombination break point analysis was used to determine that the chirality locus, if not also the gene, must be contained between markers b6 and b12 (Figure 1b). The region between these two markers contains six genes:

1. Lysosomal pro-x carboxypeptidase, *Lprcp*, 439 amino acids, three synonymous changes and zero non-synonymous changes between the dextral and sinistral haplotypes.
2. Furry, *Lfry*, 3148 amino acids, four synonymous changes and one non-synonymous change (E3082G) between the dextral and sinistral haplotypes.
3. A major facilitator superfamily domain-containing protein, *Lmfsd*, 611 amino acids, zero synonymous changes and one non-synonymous change between the dextral and sinistral haplotypes (T432S).
4. A tudor domain-containing protein, *Ltud*, 796 amino acids, two synonymous changes and six non-synonymous changes (Y119N, A307G, D327E, M367N, L375P, L503I) between the dextral and sinistral haplotypes.
5. A diaphanous-related formin, *Ldia1*, 1101 amino acids, two synonymous changes and zero non-synonymous changes between the dextral and sinistral haplotypes.
6. A diaphanous-related formin, *Ldia2*, 1047 amino acids, five synonymous changes, one non-synonymous change (D211E) and a 1 bp frame shift (L19X) between the dextral and sinistral haplotypes.

While formal proof of function is lacking, at least in snails, there is compelling evidence that *Ldia2* is a chirality-determining gene. First, the frameshift mutation, combined with a corresponding lack of transcripts in single cell embryos (see Figure 1 and qRT-PCR section below), is congruent with both maternal expression of the trait and dextral dominance. Second, while previous experiments [S7] have shown a general effect of actin disassembly upon chirality (e.g. using nocodazole), we showed that application of a specific formin-inhibiting drug mimics the sinistral phenotype (Figure 2). Differences in effect between the SMIFH2 application and the *Ldia2*^{sin} mutation are likely due to the wider action of the drug (against all FH2 domain proteins – e.g. *Inf*, *Fhod*, *Daam* are all present in the draft genome sequence) and also incomplete penetrance of the drug into the embryo.

As corroborating evidence, the general involvement of a cytoskeletal gene in determining asymmetry is expected [S28, S29]; more specifically, the tandem duplication of the formin is congruent with an earlier prediction that the locus (in *L. peregra*) is made up of more than one component or copy [32]. Finally, there is a suggestion that the ancestral Lymnaeid might have been sinistral (Figure S2c) – it makes sense that if gene duplication enabled the ancestral Lymnaeid to become dextral, then a frameshift mutation in the derived copy might make them sinistral again.

In comparison, the evidence for other candidate genes is lacking. *Ldia1* does not have any coding sequence changes between the dextral and sinistral haplotype, nor any differences in expression (Figure 1). *Lfry* is perhaps the next best candidate, since furry-like genes are microtubule-associated proteins, involved in the control of chromosome alignment, spindle organization and other activities associated with mitosis [S30]. One amino acid difference was discovered between the dextral and sinistral-derived versions of this long protein. This change is not likely to be disabling, so is incongruent with the sinistral phenotype. In addition, no differences in *Lfry* expression were discovered between dextrals and sinistrals (see below). Lysosomal pro-x carboxypeptidase proteins cleave C-terminal amino acids linked to proline in peptides such as angiotension. There are no amino acid changes between the dextral and sinistral-derived versions of *Lprcp* and no differences in maternal expression were discovered. Except for the short tudor domain (~50 amino acids; sometimes found in proteins that localise with ribonucleoprotein or single-strand DNA-associated complexes in the nucleus, in the mitochondrial membrane, or at kinetochores), the function of *Ltud* is not clear. Originally, it was thought that tudor domains might function in RNA metabolism, but it has more recently been proposed that tudor domain bind proteins with methylated amino acids, performing functions in mechanisms as diverse as snRNP metabolism or sensing of DNA double strand breaks [S31, S32]. In *L. stagnalis*, *Ltud* may be rapidly evolving, and possibly under selection, showing two synonymous changes versus six non-synonymous changes between haplotypes. In comparison with the *B. glabrata* homologue, these changes are all in highly variable (and frequently unalignable) parts of the gene. No differences in maternal expression were discovered. Major facilitator superfamily domain-containing proteins are involved in transmembrane transport of specific molecules. Only one amino acid change was discovered between the different versions of *Lmfsd* on each chromosome. No differences in maternal expression were discovered.

While the evidence is strong that the *Ldia2* is the chirality-determining gene, the chirality-region synteny between *Capitella* (Annelida) and *Lymnaea* (Mollusca) may be of functional significance. Perhaps cis-acting epistasis between two or more of the genes in this region has acted against occasional translocation events (*Ldia*, *Lfry* and *Ltud* – all involved in some aspect of mitosis?). Certainly, given that approximately half of sinistral embryos fail to hatch [S33, S34], there must be strong selection for any compensatory mutations, especially in genes that are linked to the causative gene.

qRT–PCR

Single-cell embryos of individual self-fertilised mothers were decapsulated and stored in RNAlater solution (Ambion) at 4°C. Total RNA was extracted using the RNeasy micro kit (Qiagen), yielding approximately 0.5ng total RNA per embryo. qRT-PCR was used to compare mRNA expression in single cell embryos derived from both sinistral, dextral and heterozygote mothers. The general strategy was to investigate the six candidate genes within the non-recombined haplotype (e.g. *Ldia1*, *Ldia2*, *Lfry, Lmfsd, Lprcp, Ltud*), a gene outside of the non-recombined haplotype (e.g. a FAT-like cadherin, *Lfat*), alongside several other control genes, some of which are involved in the assembly of the actin cytoskeleton (e.g. *Larp2/3* subunits 1a and 3). Primers used were: *Ldia1* 3' UTR (F: AGTGGTGTGGGCAAAAGATG; R: TATTCTGTTGATGCACGGCC), *Ldia2* 3' UTR (F: GGGAGTTCAAGTTCAAGCCTATC; R: GGCAAGCTACGACTCTTCTC), *Ldia2* ORF (F: GGGTGACAATGAAGTGGACC; R: ACATGCATCTGTAACATCTGCC), *Lfry* (F: ACTTACCCTGCTCAAATGCC; R: ATGTTTCTTGTGCTGCCGTC), *Lmfsd* (F: TGTCCTCACTGTGGCCTTTC; R: CAAACACAGGTGAAGCTGCC), *Lprcp* (F: AACTACCCTTACCCAGCGTC; R: GCTGCAAGTAGTGGTTTCCC), *Ltud* (F: TGGAGCTCAGTTTCCATTGGG; R: TTGGCCAGGCAAGTATCTCC), *Lfat* (F: TGCCCATGTTGCTAAGTTCAG; R: CCTCTATCCCAGTTCGACGG), *Larp2/3 su 1a* (F: CTGAAAATAGCCTTGTTGCAGC; R: CCAGACTCCTTTTCCTGGGAC), *Larp2/3 su 3* (F: AGCCAGCTAACAAGGGAGAAG; R: AGCATAGCCACCATTTGCTTG). Normalising control genes were *Lhis2a* (histone 2A; F: TCAGAGGAGATGAGGAGTTGG; R: CCCCAAGTTATGCTGCCTTC), *Lube2* (ubiquitin-conjugating enzyme E2; F: GCGGATCCTCTTGCAATCTT; R: TCTGTGGACTGCATATCACTCT) and *Lyhwaz* (14-3-3 protein zeta; F: GGAGGAGCTGAAGTCAATATGC; R: AGTCACCCTGCATTTTGAGG). Genes that have been frequently used by others as normalising controls, but which are involved in cytoskeletal processes (e.g. actin), were avoided.

All real-time quantitative PCR reactions were performed using SYBR green master mix and the Applied BiosystemsTM 7500 Real Time PCR System. cDNA sequence-specific primers were designed in-house and primer efficiencies were estimated from a minimum of two successful standard curve experiments. Primer specificity has supported by the presence of discrete temperature melt curves and additional gel electrophoresis and Sanger sequencing of cDNA amplified products. Normalised relative quantities (NRQs) were calculated for all experiments [S35], calibrated to a *DD* individual and normalised to three endogenous control genes, which had been verified as most stable within each tissue via geNorm [S36], NormFinder [S37], and BestKeeper [S38]. Significant differential gene expression was determined using the Wilcoxon-Mann-Whitney test, available in the standard R package.

Boxplots (Figure S3a) show normalised relative quantities (NRQ; logarithmic scale) of qRT-PCR assays of transcripts from five genes, using RNA from single cell embryos (n=17; 6 *DD*, 5 *Dd*, 6 *dd*). As the coding sequence of *Ldia2* is very similar to *Ldia1,* assays were designed for both the 3' untranslated region (UTR), to ensure primer specificity, and also the open reading frame (ORF). Overall there were very large and significant differences in expression of *Ldia2* between dextral, sinistral and heterozygote embryos (Figure 1; Figure S3a). No significant differences in expression were discovered for any of the other five candidate genes, *Ldia1*, *Lfry*, *Lmfsd*, *Lprcp*, and *Ltud*. Small but significant differences in expression were also detected in some *Larp2/3* comparisons. As *Larp2/3* is not a candidate gene, the biological significance of these differences, if any, is not clear.

Whole mount *in situ* hybridisation

We examined the spatial expression of mRNAs in early *L. stagnalis* embryos, using whole mount *in situ* hybridization (WMISH). The following is a summary of the protocol, described previously [S39, S40]. Freshly deposited egg strings were carefully collected from the walls of aquaria maintained at 23°C. Egg capsules were removed from the egg string, and as much of the jelly material removed as possible from each capsule by pushing them across a paper towel using featherweight forceps. Egg capsules were then transferred into a sealable tube that provided 10 times the volume of collected egg capsules. As much liquid as possible was aspirated from the tube and replaced with a 10 fold volume of 3.7% formaldehyde in 1 x PBS. Egg capsules were gently agitated in fixative at room temperature for 2 hours, and then washed three times for 5 minutes each in PBS + Tween 20 (PBTw). Embryos were removed from their capsules using a custom made device (see [S40]). This can also be achieved manually using fine forceps. Decapsulated embryos and larvae were then collected using a micropipette, and stepped into hybridisation buffer and allowed to pre-hybridise for 30 minutes at 55°C in 1.5 mL tubes. During this time dilutions of ribo-probes were prepared in hybridisation buffer. All ribo-probes were used at approximately 1 ng/μL. Ribo-probes were added to the samples which were then heated to 75°C for 10 minutes, and then hybridised at 55°C for 1-2 days. During this time tubes were occasionally gently agitated.

We used a liquid handling robot for the following wash steps (InsituPro VSi), however these can also easily be done manually. In this case, embryos and larvae should be kept in the 1.5 mL tubes they were hybridized in. All subsequent solution exchanges are aspirated and added with a P1000 pipette. When performed manually, each of the following washes should employ 1 mL of each solution. The 4 x, 2 x and 1 x wash solutions were pre-warmed to 55°C. Samples were washed three times in 4 x wash buffer, three times in 2 x wash buffer, and three times in 1 x wash buffer for 15 min each at 55°C. Samples were then washed once with 1 x SSC + 0.1% Tween at 55°C, and then allowed to cool to room temperature. All samples were then washed twice in 1 x SSC + 0.1% Tween for 15 min at room temperature, and then stepped into Maleic Acid Buffer (MAB) with two 10 minute washes. The MAB solution was then replaced with block solution and incubated for 1.5 hours. The block solution was then exchanged for anti-DIG antibody solution (1:10,000 dilution of the antibody in block solution) and incubated for 12 hours (overnight) at room temperature with gentle agitation.

The antibody solution was then aspirated and samples were washed 15 times with PBTw for 10 min each. PBTw was then replaced with 1 x Alkaline Phosphatase Buffer (AP) and the samples incubated for 10 min. Samples were then transferred into a well of a 12-well tissue culture dish, and the 1 x AP solution replaced with staining solution, and the development of the colour reaction was monitored. Colour development was stopped by replacing the staining solution with two exchanges of PBTw. Samples were post-fixed in 3.7% formaldehyde in PBTw and agitated for 30 min at room temperature (this was also done overnight). The fixative was washed out with 3 washes of PBT, and the samples washed 3 times at 50°C in de-ionized water to remove all residual salts before being dehydrated through an ethanol series. Samples were then mounted in benzyl benzoate: benzyl alcohol (BB:BA) and mounted for photo-documentation using a Zeiss Axio Imager with DIC optics. Images were cropped using Adobe Photoshop and arranged using Adobe Illustrator.

Previously, we validated the general WMISH protocol, by confirming the maternal expression of a variety of *L. stagnalis* gene transcripts [S41]. To validate the maternal expression of the *Ldia* and the main other

candidate gene, *Lfry*, we prepared a riboprobe based on knowledge of the full length cDNA transcript sequences – unfortunately, it was not possible to generate a probe specific to *Ldia2,* because of cross-reactivity of the probe to *Ldia1* (sequence similarity is ~90%).

The expectation was that transcripts would be distributed more or less equally between the four macromeres, as the existing dogma is that the four macromeres that are formed from the first two cleavages are indistinguishable, until contact between the third quartet of micromeres (fifth/sixth cleavage) induces one of the macromeres to become the future D blastomere, ultimately serving as an organiser of the dorso-ventral axis [S42, S43, S44]. Surprisingly, however, we found that while formin mRNA is distributed more or less evenly throughout the single cell dextral embryo, by the two cell stage it is mostly confined to one of the two macromeres, and by the four cell stage is mostly present in only one of the four macromeres (Figure 1d and S3b). In comparison, in sinistral embryos, formin staining was more variable and less obviously asymmetric. The staining of *Lfry* transcripts was not consistent and showed considerably more variation (Figure S3b).

These results therefore imply that the existing model of D quadrant specification in equal cleaving molluscs is incorrect, as individual macromeres have an identity at the four cell stage. However, while this is an interesting and exciting discovery, considerable further work will be necessary to reconcile this new data with the existing model of D quadrant specification. Moreover, it is not possible to know at this stage whether the asymmetric expression of RNA at the 4 cell stage is at all related to the chiral twist that takes place during the third cleavage. Indeed, the model does not require that an F-molecule is distributed asymmetrically. In the case of *L. stagnalis*, we need to know if the embryo is relying upon pre-existing formin protein to direct the chiral twist, or whether protein synthesis in the early embryo is involved.

Pharmacology – pond snails

We used a small molecule inhibitor of formin homology 2 domains (SMIFH2) to inhibit formin-guided actin assembly [S45]. As the name suggests, SMIFH2 targets the conserved FH2 domain, preventing both formin nucleation and processive barbed end elongation, and is of proven wide effect, being functional in yeast, nematode, mouse [S45] and *Arabidopsis* [S46]. As a comparison and control treatment, we used CK-666 (of effect in humans, mouse and yeast [S47]), an inhibitor of Arp2/3-complex mediated actin assembly. This drug is believed to act by targeting an opening between Arp2 and Arp3, preventing the complex from shifting into an active conformation [S48]. Critically, low micromolar concentrations of SMIFH2 disrupt formin-dependent, but not Arp2/3 complex-dependent, actin cytoskeletal structures [S45] – SMIFH2 is largely independent in its effect upon actin chain assembly.

We focussed efforts on understanding the effect of these drugs on the chiral twist that takes place during the 4 to 8-cell transition. Fresh embryos were dissected out of egg capsules and transferred to standard snail water [S49] in embryo dishes, where they remained until they reached the 4-cell stage. Thereafter, embryos were transferred into control dishes, or drug-containing dishes, immediately upon reaching the 4-cell stage, or 10, 30, 45 or 60 minutes later. Embryos were then observed until about mid-way (see below for specific details) through the third cleavage (~80-90 minutes after the second cleavage at 20 to 25°), at which point they were fixed. Embryos were imaged under a Nikon SMZ1500 microscope with DXM1200F camera. The rotation of each micromere was estimated, by measuring the angle produced by two lines, one that bisects the macromere and one that bisects the micromere, using ImageJ [S50]. For purposes of analysis, we defined a "neutral" emergence as an angle of ±5° – although this definition is certainly arbitrary, a more stringent definition still produced significant numbers of neutral embryos after SMIFH2 treatment.

Ordinarily, the first quartet of micromeres in a dextral embryo simultaneously emerge *and* turn at the same time, so that by the time they have fully emerged, the angle of turn approaches +35-45°. In contrast, in sinistral embryos the micromeres emerge first, then turn during furrow ingression [S7]. As we wished to measure the angle of rotation upon first emergence, not the final angle, individual embryos were observed and fixed as the micromeres first emerged, where "emerged" is defined as showing significant furrow ingression, such that the micromere is a defined and separate body from the macromere. Occasionally, not all four micromeres emerged at the same time, in which case embryos were fixed as soon as three micromeres had emerged – if we had waited until all four had emerged then the first three would have turned further.

SMIFH2 and CK-666 (both 100 µM final concentration, in DMSO; Sigma S4826, SML0006) were separately added to decapsulated 4-cell embryos, immediately after second cleavage had completed. Embryos were fixed during the third cleavage, as the micromeres emerged, and the angle of rotation scored qualitatively. In an initial experiment, the chiral twist was scored qualitatively (sinistral, dextral or neutral). In control and drug treated experiments, the majority of genetically dextral embryos twisted dextrally (84.7% control, n=150; 56.0% SMIFH2, n=150; 81.3% CK-666; n=150). Nonetheless, 28% of SMIFH2-treated embryos, 12.7% of CK-666-treated embryos and 4.7% of the controls emerged without rotation. The remainder of the embryos showed partial rotation, or were difficult to score. In comparison, the majority of genetically sinistral embryos emerged

without a chiral twist, irrespective of whether they had been drug treated or not (98.0% control, n=163; 98.0 % SMIFH2, n=152; 94.8% CK-666; n=134). The procedure was then repeated using a quantitative approach. Broadly, the results were the same between both methods: SMIFH2 has the greatest impact upon the chiral twist of emergence, with CK-666 having a lesser impact (Figure 2; Table S2).

To understand the wider significance of these results, we attempted to carry out the same experiment in sinistral-cleaving *B. glabrata*. Unfortunately, SMIFH2 treatment was invariably lethal even when added just before the third cleavage. Thus, while formins are certainly implicated in early development of *B. glabrata*, we do not know if they are implicated in directing the chiral twist at third cleavage.

For microscopy, embryos were fixed in 4% paraformaldehyde and incubated in blocking buffer (2% foetal calf sera, 0.1% Triton-X-100) before immunostaining with alexa488-phalloidin (Life Technologies) and Cy3-β-tubulin (Sigma). Confocal microscope images were acquired using a Leica TCS-NT confocal microscope with a 60x/1.2 numerical aperture objective. Images were processed with Huygens Deconvolution Software (Scientific Volume Imaging, Hilversum, Netherlands) and Adobe Photoshop.

Pharmacology and microinjection – *Xenopus*

Embryos were treated at the stages indicated with either DMSO alone, CK-666 in DMSO at the final concentration indicated (Sigma, SML0006) or SMIFH2 in DMSO at the final concentration indicated (ChemBridge Corporation, 5992446). Embryos were washed with at least 5 washes of 0.1 x Modified Marc's Ringers (MMR), pH 7.8 after treatment and raised to Stage 45 [S1].

Xenopus embryos were analyzed for position (*situs*) of three organs; the heart, stomach and gallbladder [S51] at Stage 45 [S1]. Heterotaxic embryos were defined as having a reversal in one or more organs. Only embryos with normal dorsoanterior development and clear left- or right-sided organs were scored. Percent heterotaxia was calculated as the number of heterotaxic embryos divided by the number of total scorable embryos. A X^2 test was used to compare absolute counts of heterotaxic embryos compared to control untreated embryos (Figure 4; Table S3).

For microinjection, capped, synthetic mRNAs encoding mouse *diaphanous1* were dissolved in water and injected into embryos in 3% ficoll using standard methods [S12]. mRNA injections were made into the animal pole of eggs at the timepoints indicated using borosilicate glass needles calibrated to deliver a 10 nl injection volume.

Phylogenetics

Previously, Chalkia *et al.* (2008) used an alignment of the FH2 domain to understand the evolutionary relationship between formins from diverse prokaryotic and eukaryotic genomes [S52]. To better understand the relationship between *Ldia1, Ldia2* and other formins, we included our new sequences in the same FH2 alignment. To put the pond snail sequences into context, we also mined genome and transcriptome databases to derive putative *dia* gene sequences for *L.* (*Galba*) *trunculata* [S53], *Physa acuta, B. glabrata, Lottia gigantea, Crassostrea gigas* (all molluscs) and *Capitella telata* (an annelid). The most appropriate model of evolution was identified using Prottest 3.4 [S54] then a maximum likelihood phylogeny was estimated in PhyML [S55]. Trees were then bootstrapped using 100 replicates and visualised using TreeExplorer (Figure S2a). To examine further examine the relationship of *dia* genes within the molluscan group alone, we identified putative orthologues by tblastn of Genbank Transcriptome Shotgun Assembly, as well as genome specific databases. Following alignment using Muscle, an appropriate model of evolution was selected using jModelTest and the Akaike Information Criterion [S56]; followed by tree construction and visualisation using PhyMl and TreeExplorer, including bootstrap support (Figure S2b). A phylogeny was also produced using MrBayes 3.2.5 [S57].

In order to investigate associations between variation in the *dia* gene sequence and chirality in other species, we used intron-spanning primers (described above) to generate sequence from two land snail genera, *Euhadra* and *Partula*. In *Euhadra,* there was considerable variation between species. Using similar methods as above, a phylogeny was constructed (Figure S2d) and chirality mapped onto it. For *Partula*, less variation was present, so a NeighbourNet network, based on a matrix of uncorrected p-distances using SplitsTree 4.12 [S58], was instead constructed (Figure S2e).

In the most recent comprehensive revision of the taxonomy of gastropod molluscs, it was proposed that a "Hygrophila" clade contains six families, the dextral Chilinidae, Latiidae, Acroloxidae, Lymnaeidae and the sinistral Planorbidae and Physidae [S59]. To date, no study has been able to uncover the precise relationship between these groups, and thus the evolution of chirality, either because of incomplete sampling of representatives from each family [S60] or poor statistical support for key branches [S61, S62]. For example, it was shown that representatives of the Acroloxidae, Lymnaeidae, Planorbidae and Physidae form a monophyletic group, though the topology was unresolved [S61]. In another study, representatives from the Physidae and

Lymnaeidae were shown to cluster together, separately from the Planorbidae, but the Chilinidae and Acroloxidae were not sampled [S60]. Lastly, Klussmann-Kolb *et al.* sampled all six families, recovering a monophyletic Hygrophila group, but statistical support for the internal branches was low [S62] (e.g. the relationship between the Lymnaeida, Physidae and Planorbidae was not clear – Bayesian posterior probabilities <<0.95, some branches not supported at all with ML).

To test the monophyly of the Hygrophila clade, to resolve the relationships within the group, and to understand the evolution of chirality, we produced a genus-level phylogeny containing representatives from all six families, combining existing ~1.7 kb 18S sequences, with newly generated ~3.5 kb 28S rRNA sequences [S59, S61] using previously described primers [S63] and new primers: set C (LSU-4 GTCGGCATTCCACCCGACC, LSU-7 GCAGGTGAGTTGTTACACACTC), Set D (LSU-6 GTGCCAAACGCTGACGCTCA, LSU-9 ACCCAGTCCTCAGAGCCAATC), Set E (LSU-8 CACAGCCTCTAGTCGATAGAAC, LSU-11 CCGTCCTCCTGAGCTCGCC), Set F (LSU-10 GGCCGCGATCCGTCTGAAGA, LSU-12 GGCTTCTGACTTAGAGGCGTT). The 28S rRNA sequence was combined with ~1.7 kb of existing 18S rRNA sequences to produce a genus-level phylogeny, selecting an appropriate model of evolution using jModelTest and the Akaike Information Criterion [S56]; followed by tree construction and visualisation using PhyMl and TreeExplorer, including bootstrap support (Figure S2c). Phylogenies were also produced using MrBayes 3.2.5 [S57].

The resulting phylogeny (Figure S2c) confirms that the proposed Hygrophila grouping is monophyletic, with strong statistical support. The phylogeny also shows, with strong statistical support, that representatives of the Physidae and Lymnaeaidae cluster together (congruent with the study of Holznagel *et al.*, also using 28S sequences but different taxa). By mapping chirality onto the phylogeny, we infer that dextrality is most likely the ancestral condition within the group. Subsequently, either sinistrality evolved once, with a subsequent reversion to dextrality in the Lymnaeidae, or else sinistrality evolved on two separate occasions, in the Physidae and the Planorbidae. Both explanations are equally parsimonious.

As the above inference is critically dependent upon the tree topology, we compared the best tree (topology: [Planorbidae, [Physidae, Lymnaeaidae]]) against a tree constrained such that the sinistral Physidae and Planorbidae are monophyletic (topology: [Lymnaeaidae, [Planorbidae, Physidae]]). Using a relatively conservative Shimodaira-Hasegawa test [S64], the alternative topology provides a significantly worse explanation of the data (P=0.043).

The accessions for this work (new accessions all begin with 'KU') are:

Basommatophora

Siphonaria pectinata (HQ659934; KU341320).

Basommatophora, clade Hygrophila

Acroloxidae: *Acroloxus lacustris* (AY282592; KU341306).

Chilinidae: *Chilina sp.* (EF489358; HQ659964).

Latiidae: *Latia neritoides* (EF489339; FJ917245, AY465072 and EF489359).

Lymnaeidae: *Lymnaea stagnalis* (EF489345; KU341307), *Radix auricularia* (FR797817; AY465067).

Physidae: *Aplexa sp.* (FR797814; AY465071); *Physa sp.* (AY282600; KU341308).

Planorbidae: *Biomphalaria glabrata* (U65223; AF435694 and contig18577, genome assembly 4.3), *Ferrissia sp.* (AY282597; KU341310), *Glyptophysa gibbosa* (EF012196; DQ256736), *Planorbis planorbis* (EF012192; KU341309).

Eupulmonata

Laemodonta sp. (HQ659951; KU341316), *Melampus sp.* (HQ659940; KU341317), *Carychium sp.* (EF489341; KU341318).

Eupulmonata, clade Systellommatophora

Laevicaulis altae (X94270; KU341319).

Eupulmonata, clade Stylommatophora

Arion sp. (HQ659992; KU341315), *Cepaea nemoralis* (AJ224921; KU341312), *Cochlicopa sp.* (GU331944; KU341313), *Deroceras reticulatum* (AY145373; KU341314). *Limicolaria kambeul* (X66374; KU341311).

Opisthobranchia (outgroup) *Aplysia sp.* (AY039804; KU341321).

Supplemental References

S1. Nieuwkoop, P.D., and Faber, J. (1994). Normal Table of *Xenopus Laevis* (Daudin), 1st Edition, (New York: Garland Science).

S2. Boycott, A.E., and Diver, C. (1923). On the inheritance of sinistrality in *Limnaea peregra*. Proc. R. Soc. Biol. Sci. Ser. B *95*, 207-213.

S3. Zimmerman, E.C. (1948). Insects of Hawaii, Volume 1, (Honolulu: University of Hawaii Press).

S4. Pierce, H.G. (1996). On sinistral coiling among fossil North American Lymnaeidae. Veliger *39*, 220-225.

S5. Asami, T., Gittenberger, E., and Falkner, G. (2008). Whole-body enantiomorphy and maternal inheritance of chiral reversal in the pond snail *Lymnaea stagnalis* J. Hered. *99*, 552-557.

S6. Kuroda, R., Endo, B., Abe, M., and Shimizu, M. (2009). Chiral blastomere arrangement dictates zygotic left-right asymmetry pathway in snails. Nature *462*, 790-794.

S7. Shibazaki, Y., Shimizu, M., and Kuroda, R. (2004). Body handedness is directed by genetically determined cytoskeletal dynamics in the early embryo. Curr. Biol. *14*, 1462-1467.

S8. Davison, A., Frend, H.T., Moray, C., Wheatley, H., Searle, L.J., and Eichhorn, M.P. (2009). Mating behaviour in *Lymnaea stagnalis* pond snails is a maternally inherited, lateralised trait. Biol. Lett. *5*, 20-22.

S9. Liu, M.M., Davey, J.W., Banerjee, R., Han, J., Yang, F., Aboobaker, A., Blaxter, M.L., and Davison, A. (2013). Fine mapping of the pond snail left-right asymmetry (chirality) locus using RAD-Seq and Fibre-FISH. PLOS One *8*, e71067.

S10. Davison, A., Chiba, S., Barton, N.H., and Clarke, B.C. (2005). Speciation and gene flow between snails of opposite chirality. PLoS Biology *3*, e282.

S11. Clarke, B., and Murray, J. (1969). Ecological genetics and speciation in land snails of the genus *Partula*. Biol. J. Linn. Soc. *1*, 31-42.

S12. Sive, H.L., Grainger, R.M., and Harland, R.M. (2000). Early development of *Xenopus laevis*: A laboratory manual.

S13. Thiel, T., Kota, R., Grosse, I., Stein, N., and Graner, A. (2004). SNP2CAPS: a SNP and INDEL analysis tool for CAPS marker development. Nucleic Acids Res. *32*.

S14. Martin, M. (2011). Cutadapt removes adapter sequences from high-throughput sequencing reads. EMBnet Journal *17*, 10-12.

S15. Bolger, A.M., Lohse, M., and Usadel, B. (2014). Trimmomatic: a flexible trimmer for Illumina sequence data. Bioinformatics *30*, 2114-2120.

S16. Kelley, D.R., Schatz, M.C., and Salzberg, S.L. (2010). Quake: quality-aware detection and correction of sequencing errors. Genome Biol *11*, R116.

S17. Luo, R., Liu, B., Xie, Y., Li, Z., Huang, W., Yuan, J., He, G., Chen, Y., Pan, Q., Liu, Y., et al. (2012). SOAPdenovo2: an empirically improved memory-efficient short-read *de novo* assembler. Gigascience *1*, 18.

S18. Zhang, J., Kobert, K., Flouri, T., and Stamatakis, A. (2014). PEAR: a fast and accurate Illumina Paired-End reAd mergeR. Bioinformatics *30*, 614-620.

S19. Langmead, B., and Salzberg, S.L. (2012). Fast gapped-read alignment with Bowtie 2. Nat. Methods *9*, 357-359.

S20. Bankevich, A., Nurk, S., Antipov, D., Gurevich, A.A., Dvorkin, M., Kulikov, A.S., Lesin, V.M., Nikolenko, S.I., Pham, S., Prjibelski, A.D., et al. (2012). SPAdes: a new genome assembly algorithm and its applications to single-cell sequencing. J. Comput. Biol. *19*, 455-477.

S21. Holt, C., and Yandell, M. (2011). MAKER2: an annotation pipeline and genome-database management tool for second-generation genome projects. BMC Bioinformatics *12*, 491.

S22. Stanke, M., Keller, O., Gunduz, I., Hayes, A., Waack, S., and Morgenstern, B. (2006). AUGUSTUS: ab initio prediction of alternative transcripts. Nucleic Acids Res. *34*, W435-439.

S23. Li, H., Handsaker, B., Wysoker, A., Fennell, T., Ruan, J., Homer, N., Marth, G., Abecasis, G., Durbin, R., and Genome Project Data Processing, S. (2009). The Sequence Alignment/Map format and SAMtools. Bioinformatics *25*, 2078-2079.

S24. Garrison, E., and Marth, G. (2012). Haplotype-based variant detection from short-read sequencing. In ArXiv e-prints, Volume 1207. p. 3907.

S25. Slater, G., and Birney, E. (2005). Automated generation of heuristics for biological sequence comparison. BMC Bioinformatics *6*, 31.

S26. Quinlan, A.R. (2014). BEDTools: The Swiss-Army Tool for Genome Feature Analysis. Curr. Protoc. Bioinformatics *47*, 11 12 11-11 12 34.

S27. Milne, I., Stephen, G., Bayer, M., Cock, P.J.A., Pritchard, L., Cardle, L., Shaw, P.D., and Marshall, D. (2013). Using Tablet for visual exploration of second-generation sequencing data. Briefings in Bioinformatics *14*, 193-202.

S28. Tee, Y.H., Shemesh, T., Thiagarajan, V., Hariadi, R.F., Anderson, K.L., Page, C., Volkmann, N., Hanein, D., Sivaramakrishnan, S., Kozlov, M.M., et al. (2015). Cellular chirality arising from the self-organization of the actin cytoskeleton. Nat. Cell Biol. *17*, 445-457.
S29. Mogilner, A., and Fogelson, B. (2015). Cytoskeletal chirality: swirling cells tell left from right. Curr. Biol. *25*, R501-R503.
S30. Nagai, T., and Mizuno, K. (2014). Multifaceted roles of furry proteins in invertebrates and vertebrates. Journal of Biochemistry *155*, 137-146.
S31. Thomson, T., and Lasko, P. (2005). Tudor and its domains: germ cell formation from a Tudor perspective. Cell Res. *15*, 281-291.
S32. Cote, J., and Richard, S. (2005). Tudor domains bind symmetrical dimethylated arginines. J. Biol. Chem. *280*, 28476-28483.
S33. Davison, A., Barton, N.H., and Clarke, B. (2009). The effect of coil phenotypes and genotypes on the fecundity and viability of *Partula suturalis* and *Lymnaea stagnalis*: implications for the evolution of sinistral snails. J. Evol. Biol. *22*, 1624-1635.
S34. Utsuno, H., Asami, T., Van Dooren, T.J.M., and Gittenberger, E. (2011). Internal selection against the evolution of left-right reversal. Evolution *65*, 2399-2411.
S35. Pfaffl, M.W. (2001). A new mathematical model for relative quantification in real-time RT-PCR. Nucleic Acids Res. *29*.
S36. Vandesompele, J., De Preter, K., Pattyn, F., Poppe, B., Van Roy, N., De Paepe, A., and Speleman, F. (2002). Accurate normalization of real-time quantitative RT-PCR data by geometric averaging of multiple internal control genes. Genome Biology *3*, research0034.0031 - research0034.0011.
S37. Andersen, C.L., Jensen, J.L., and Orntoft, T.F. (2004). Normalization of real-time quantitative reverse transcription-PCR data: A model-based variance estimation approach to identify genes suited for normalization, applied to bladder and colon cancer data sets. Cancer Res. *64*, 5245-5250.
S38. Pfaffl, M.W., Tichopad, A., Prgomet, C., and Neuvians, T.P. (2004). Determination of stable housekeeping genes, differentially regulated target genes and sample integrity: BestKeeper - Excel-based tool using pair-wise correlations. Biotechnol. Lett. *26*, 509-515.
S39. Hohagen, J., Herlitze, I., and Jackson, D.J. (2015). An optimised whole mount in situ hybridisation protocol for the mollusc Lymnaea stagnalis. BMC Dev. Biol. *15*.
S40. Jackson, D.J., Herlitze, I., and Hohagen, J. (2015). A whole mount *in situ* hybridization method for the gastropod mollusc *Lymnaea stagnalis*. Journal of Visualized Experiments *in press*.
S41. Liu, M.M., Davey, J.W., Jackson, D.J., Blaxter, M.L., and Davison, A. (2014). A conserved set of maternal genes? Insights from a molluscan transcriptome. Int. J. Dev. Biol. *58*, 501-511.
S42. Freeman, G., and Lundelius, J.W. (1992). Evolutionary Implications of the Mode of D-Quadrant Specification in Coelomates with Spiral Cleavage. J. Evol. Biol. *5*, 205-247.
S43. Martindale, M.Q. (1986). The organizing role of the D quadrant in an equal-cleaving spiralian, *Lymnaea stagnalis* as studied by UV laser deletion of macromeres at intervals between 3rd and 4th quartet formation. International Journal of Invertebrate Reproduction and Development *9*, 229-242.
S44. Lambert, J.D., and Nagy, L.M. (2002). Asymmetric inheritance of centrosomally localized mRNAs during embryonic cleavages. Nature *420*, 682-686.
S45. Rizvi, S.A., Neidt, E.M., Cui, J., Feiger, Z., Skau, C.T., Gardel, M.L., Kozmin, S.A., and Kovar, D.R. (2009). Identification and characterization of a small molecule inhibitor of formin-mediated actin assembly. Chem. Biol. *16*, 1158-1168.
S46. Rosero, A., Zarsky, V., and Cvrckova, F. (2013). AtFH1 formin mutation affects actin filament and microtubule dynamics in *Arabidopsis thaliana*. J. Exp. Bot. *64*, 585-597.
S47. Nolen, B.J., Tomasevic, N., Russell, A., Pierce, D.W., Jia, Z., McCormick, C.D., Hartman, J., Sakowicz, R., and Pollard, T.D. (2009). Characterization of two classes of small molecule inhibitors of Arp2/3 complex. Nature *460*, 1031-1034.
S48. Hetrick, B., Han, M.S., Helgeson, L.A., and Nolen, B.J. (2013). Small Molecules CK-666 and CK-869 Inhibit Actin-Related Protein 2/3 Complex by Blocking an Activating Conformational Change. Chem. Biol. *20*, 701-712.
S49. Thomas, J.D. (1986). The Chemical Ecology of *Biomphalaria glabrata* (Say) - Sugars as attractants and arrestants. Comp. Biochem. Physiol. A: Physiol. *83*, 457-460.
S50. Schneider, C.A., Rasband, W.S., and Eliceiri, K.W. (2012). NIH Image to ImageJ: 25 years of image analysis. Nat. Methods *9*, 671-675.
S51. Levin, M., and Mercola, M. (1998). Gap junctions are involved in the early generation of left-right asymmetry. Dev. Biol. *203*, 90-105.
S52. Chalkia, D., Nikolaidis, N., Makalowski, W., Klein, J., and Nei, M. (2008). Origins and evolution of the formin multigene family that is involved in the formation of actin filaments. Mol. Biol. Evol. *25*, 2717-2733.

S53. Burgarella, C., Gayral, P., Ballenghien, M., Bernard, A., David, P., Jarne, P., Correa, A., Hurtrez-Boussès, S., Escobar, J., Galtier, N., et al. (2015). Molecular evolution of freshwater snails with contrasting mating systems. Mol. Biol. Evol.

S54. Darriba, D., Taboada, G.L., Doallo, R., and Posada, D. (2011). ProtTest 3: fast selection of best-fit models of protein evolution. Bioinformatics *27*, 1164-1165.

S55. Guindon, S., and Gascuel, O. (2003). A simple, fast, and accurate algorithm to estimate large phylogenies by maximum likelihood. Syst. Biol. *52*, 696–704.

S56. Darriba, D., Taboada, G.L., Doallo, R., and Posada, D. (2012). jModelTest 2: more models, new heuristics and parallel computing. Nat. Methods *9*, 772-772.

S57. Ronquist, F., and Huelsenbeck, J.P. (2003). MrBayes 3: Bayesian phylogenetic inference under mixed models. Bioinformatics *19*, 1572-1574.

S58. Huson, D.H., and Bryant, D. (2006). Application of phylogenetic networks in evolutionary studies. Mol. Biol. Evol. *23*, 254-267.

S59. Bouchet, P., Rocroi, J.P., Fryda, J., Hausdorf, B., Ponder, W., Valdes, A., and Waren, A. (2005). Classification and nomenclator of gastropod families. Malacologia *47*, 1-368.

S60. Holznagel, W.E., Colgan, D.J., and Lydeard, C. (2010). Pulmonate phylogeny based on 28S rRNA gene sequences: A framework for discussing habitat transitions and character transformation. Mol. Phylogenet. Evol. *57*, 1017-1025.

S61. Dayrat, B., Conrad, M., Balayan, S., White, T.R., Albrecht, C., Golding, R., Gomes, S.R., Harasewych, M.G., and de Frias Martins, A.M. (2011). Phylogenetic relationships and evolution of pulmonate gastropods (Mollusca): New insights from increased taxon sampling. Mol. Phylogenet. Evol. *59*, 425-437.

S62. Klussmann-Kolb, A., Dinapoli, A., Kuhn, K., Streit, B., and Albrecht, C. (2008). From sea to land and beyond - New insights into the evolution of euthyneuran Gastropoda (Mollusca). BMC Evol. Biol. *8,* 57..

S63. Wade, C.M., Mordan, P.B., and Naggs, F. (2006). Evolutionary relationships among the Pulmonate land snails and slugs (Pulmonata, Stylommatophora). Biol. J. Linn. Soc. *87*, 593-610.

S64. Shimodaira, H., and Hasegawa, M. (1999). Multiple comparisons of log-likelihoods with applications to phylogenetic inference. Mol. Biol. Evol. *16*, 1114-1116.
