# Supplementary figures and images for: Formin Is Associated with Left-Right Asymmetry in the Pond Snail and the Frog
Source: Curr Biol. 2016 Mar 7;26(5):654–60. doi: 10.1016/j.cub.2015.12.071 (PMC4791482; doi:10.1016/j.cub.2015.12.071)

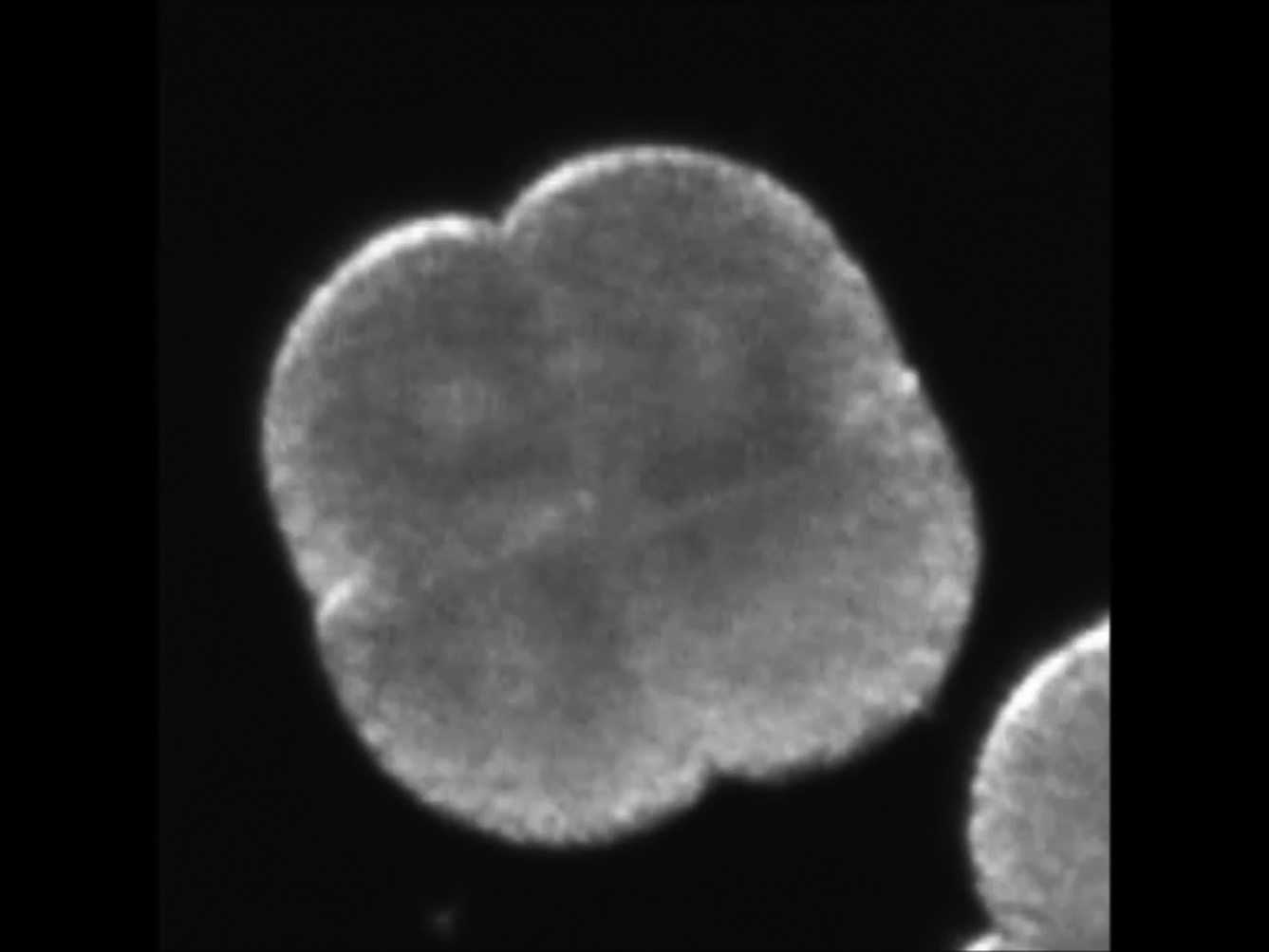

Supplement: Movie S1. Time-Lapse of Third Cleavage, Related to Figures 1 and 2 — In embryos from genetically dextral mothers, the micromeres twist dextrally as they emerge. In embryos from genetically sinistral mothers, the micromeres emerge neutrally, then twist sinistrally after emergence. In SMIFH2-treated embryos from genetically dextral mothers, the micromeres frequently emerge neutrally, then twist dextrally after emergence. Occasionally, in embryos from genetically sinistral mothers, the micromeres emerge neutrally, as normal, but the later twist is dextral. [file mmc2.jpg]
